# The Scientific Filesystem

**DOI:** 10.1093/gigascience/giy023

**Published:** 2018-03-13

**Authors:** Vanessa Sochat

**Affiliations:** 1 Stanford Research Computing Center; 2 Stanford University School of Medicine, Stanford, CA 94025

**Keywords:** filesystem, reproducibility, singularity, hpc, workflows, Linux containers, Docker, containers

## Abstract

**Background:**

Here, we present the Scientific Filesystem (SCIF), an organizational format that supports exposure of executables and metadata for discoverability of scientific applications. The format includes a known filesystem structure, a definition for a set of environment variables describing it, and functions for generation of the variables and interaction with the libraries, metadata, and executables located within. SCIF makes it easy to expose metadata, multiple environments, installation steps, files, and entry points to render scientific applications consistent, modular, and discoverable. A SCIF can be installed on a traditional host or in a container technology such as Docker or Singularity. We start by reviewing the background and rationale for the SCIF, followed by an overview of the specification and the different levels of internal modules (“apps”) that the organizational format affords. Finally, we demonstrate that SCIF is useful by implementing and discussing several use cases that improve user interaction and understanding of scientific applications. SCIF is released along with a client and integration in the Singularity 2.4 software to quickly install and interact with SCIF. When used inside of a reproducible container, a SCIF is a recipe for reproducibility and introspection of the functions and users that it serves.

**Results:**

We use SCIF to evaluate container software, provide metrics, serve scientific workflows, and execute a primary function under different contexts. To encourage collaboration and sharing of applications, we developed tools along with an open source, version-controlled, tested, and programmatically accessible web infrastructure. SCIF and associated resources are available at https://sci-f.github.io. The ease of using SCIF, especially in the context of containers, offers promise for scientists’ work to be self-documenting and programatically parseable for maximum reproducibility. SCIF opens up an abstraction from underlying programming languages and packaging logic to work with scientific applications, opening up new opportunities for scientific software development.

## Conclusions

- Software, environments, and metadata for scientific applications are not easily exposed.
- Inspection and predictability of applications are essential for scientific reproducibility
- SCIF makes it easy to generate modular, predictable, and programmatically understandable scientific applications.

## Introduction

For quite some time, our unit of understanding computer systems has been based on the operating system. It is the level of magnification at which we operate (personal computers) and the starting point for working with data, software, and services [1]. With the increasing need to define, utilize, inspect, and transport scientific applications, we are faced with the dual need to install said applications alongside a host while not being confused or lost within it. The rising popularity of Linux containers [2–5] helped greatly to expose scientific applications, as a container could be specialized to perform 1 task. While this practice affords reproducibility by way of providing encapsulated, portable environments, in the same way that we cannot reliably predict how to call a program on a colleague’s computer without having outside knowledge, the contents of the containers are not internally modular or programmatically understandable. Even with a new focus on using containers, the reproducibility of an operating system does not guarantee its discoverability. Whether installed on a host or within a container, it tends to be the case that scientific applications, without special knowledge from the creator, are akin to black boxes. For the scientific community, this is a barrier to reproducibility.

If the goal is scientific reproducibility, we can start our discussion with an example of a modern day best practice for reproducibility: a scientific application installed alongside an operating system in a container. How might we interact with this black box container? In the best case scenario, executing the container will reveal instructions for usage via the container’s single, defined entry point. If the container has more than 1 executable, this model already falls short. An assessment of a container’s ability to fully communicate its usage can start with a series of simple questions. Can the executables for the analysis be predictably found? If a particular directory is mounted as a volume from the host, is any important container content lost due to overlay? Can we untangle the scientist’s contributions from the base operating system? Without a consistent approach to ensure internal consistency and modularity, these questions are not answered easily. The reason is because reproducibility does not guarantee an ability to introspect or discover content. A definition is needed to distinguish the scientific applications from the host filesystem and to expose executables and environments throughout the host’s life cycle [6]. Such a specification must address the following issues:

- Installation of applications on a host filesystem is not *consistent* to allow for comparison of applications generated by different individuals.
- Traditional filesystems are not *transparent*. If I discover a host with software installed and do not have any prior knowledge or metadata, a known function may be completely concealed.
- Scientific applications on a host are not programmatically *understandable*. I should be able to inspect a host, know exactly the applications available to me, ask for help, and easily be able to discover metadata and files.
- Installation of scientific applications on a host is not *modular*. We are not able to distinguish content, environment, and metadata between applications.

The base of these problems can be reduced to the fact that we are being forced to operate using an organization that no longer makes sense given the problem at hand. Furthering reproducible practices calls for optimized definition and modular organization of applications and data, and this is a different set of goals than structuring a base system per the Filesystem Hierarchy Standard [7,8]. This goal is also not met by assigning one software package via a shell such as a container, because there is huge redundancy with regard to the duplicated filesystem, and the added software is now hiding among it.

The above problems also hint that the generation of reproducible scientific applications is not easy. When a scientist starts to write a build specification^1^, he probably doesn’t know where to install software, or perhaps that a help file should exist, or that metadata about the software should be easily accessible. To generate applications that integrate with external hosts, data, and other applications, the generating software needs to easily capture this content automatically.

Based on these problems, it is clear that we need direction and guidance on how to organize multiple applications and data on a host in a way that affords modularity, programmatic accessibility, transparency, and consistency. Here, we review the rationale and use cases for the Scientific Filesystem (SCIF). We first review the goals of the architecture, followed by integrations and tools, and then the organizational standard itself. To demonstrate utility, we describe several implemented use cases in the context of containers for assessment of container metrics and running scientific workflows. The document that describes the specification for SCIF is openly available for critique and contribution at https://sci-f.github.io/, and the client at https://vsoch.github.io/scif and contributions from the community are encouraged.

## Goals

SCIF establishes an overall goal to make scientific applications consistent, transparent, parseable, and modular. SCIF brings modularity and exposure to otherwise hidden application content. In the following sections, we define a host as the operating system running on a machine or the operating system inside a container. We assert that for a host to conform to SCIF, it must:

- Be *consistent* to allow for comparison. I am able to easily discover relevant software and data for 1 or more applications defined by the creator.
- Be *transparent*. If I discover a SCIF and do not have any prior knowledge or metadata, the important executables and metadata are revealed to me.
- Make application contents easily available for introspection, meaning the application contents are programmatically *parseable*.
- Provide application organization that is *modular*. Given a set of SCIF apps from different sources, I can import different install routines and have assurance that environment variables defined for each are sourced correctly for each and that associated content does not overwrite previous content. Each software and data module must carry, minimally, a unique name and install location in the system.

To be clear, this is not a specification for a container image^2^, or a workflow manager [9–12], or a package manager [13,14]. Although these goals match nicely with efforts for workflow and image or package standardization [15], SCIF is not intended for package management or running workflows and carries more functionality than simple activation of environments or ensuring reproducibility [16]. SCIF is a specification for modular organization of content, environment variables describing it, and core functions to interact with it. As defined by the formal specification (https://sci-f.github.io/specification), a client is a controller for a SCIF, either for a developer or user, and an integration is a third-party software or tool that understands the SCIF structure and interacts with all or some portion of it. Any client that implements SCIF to achieve the goals of consistency, transparency, and modularity will provide an easy means to adopt the organizational structure defined by the specification and expose both functions and environment variables to interact with it. Any technology that integrates with SCIF should expect this organization and environment variables. For the interested reader, the formal specification and resources are available at https://sci-f.github.io, and each of the specific goals in context of the assertions is discussed in more detail in the following sections. For these goals, we introduce the idea of container “apps,” or subfolders corresponding to an internal module that conform to a predictable internal organization under /scif/apps. We refer to the general “host” as reference to a computer’s base operating system or the operating system of a container running on it. For the example commands, we demonstrate usage of our referenced client “scif” that implements functions from the specification to interact with the SCIF filesystem (/scif) and environment variables (starting with the prefix “SCIF_”).

### Consistency

Given the case of 2 hosts with the same software installed, it should be the case that the software with some unique resource identifier (commonly a name and version) and any included data can be consistently found. To achieve this goal, SCIF defines a new root folder, /scif, a name that should have minimal conflict with existing cluster resources. Under this folder are separate folders for each of the software modules, “apps,” under /scif/apps, and data under /scif/data. Under these 2 folders is where the subfolders for internally modular installed applications (apps) live. For example, a host with applications foo and bar would have them installed as follows:

\begin{array}{l}
{\rm /scif} \\ \qquad {\rm /apps}\\  \qquad \qquad {\rm /bar}\\ \qquad\qquad {\rm /foo}
\end{array}

If 2 hosts both have foo installed, we would know to find the installation under /scif/apps/foo. Data takes a similar approach. We define a new root folder, /scif/data, with a similar subfolder organization:

\begin{array}{l}
{\rm /scif}\\ \qquad {\rm /data}\\ \qquad\qquad{\rm /bar}\\ \qquad\qquad {\rm /foo}
\end{array}

A workflow that knows to execute the foo application would also give the user guidance about where to mount to interact with data, and these locations would not conflict with another application. Although SCIF is not a workflow specification or manager, the exposure of consistent data and executable locations makes it a powerful tool when paired with one.

## Transparency

Arguably, if we discover a host and want to know about the intended use of its applications, we don’t care so much about what the underlying operating system is. We would want to subtract this base first and then glimpse at what remains. Given the consistent organization above, we can easily distinguish the scientific applications from the host with a simple function required by the specification to list apps:

\begin{array}{l}
{\rm \$ \,scif \,apps}\\
\\
\hphantom{\$ \,}{\rm bar}\\
\hphantom{\$\, }{\rm  foo}
\end{array}

We can predictably find and investigate a particular software given that we now know the name. In the example below, we demonstrate that executing the entry point to a SCIF in the context of a particular application (foo) exposes important information about foo via environment variables.

\begin{array}{l}
 {\rm \$\, scif\, shell\, foo}\\
\\
{\rm \$\, echo\, \$ SCIF\_APPNAME}\\
{\rm  foo}\\
\\
{\rm \$\, echo\, \$SCIF\_APPROOT}\\
{\rm  /scif/apps/foo}\\
\\
{\rm  \$\, ls\, \$SCIF\_APPROOT}\\
{\rm  bin}\\
{\rm  lib}\\
{\rm  scif}
\end{array}

The app can also be run, asking scif to call the defined executable for the app:

\begin{array}{l}
{\rm  \$\, [foo]\, executing\, /bin/bash}\\
\qquad\qquad{\rm /scif/apps/hello-world-echo/scif/runscript}\\
{\rm  The\, best\, app\, is\, foo}
\end{array}

The uniqueness of the base path /scif is important because of mounting. For hosts that do not support overlays [17], the mount point must also exist on the host, and so the primary folders for the Standard Container Integration Format should not interfere with any that exist on the host (e.g., /opt). From a high level, we are introducing a simply formatted addition to the standard Linux File System Hierarchy, a folder at the root that starts with /scif that makes an assertion that the content under this base is exclusively relevant to the purpose of the container, and not the base operating system.

## Parsability

Parsability comes down to programmatic accessibility. This means that, for each software module installed, we need to be able to do the following:

- **Provide metadata.** A software module might have a version, a link to further documentation, an author list, or other important metadata values that should be programmatically accessible.
- **Provide an entry point.** Different software modules on a host should each be able to define an entry point. In the context of a container, this is incredibly salient because containers traditionally expose only one (the “runscript” for a Singularity container, or the ENTRYPOINT and CMD for a Docker container).
- **Provide help.** Given an entry point, or if a user wants to understand an installed application, it should be the case that a command can be issued to view documentation provided for the software. For the developer, adding this functionality should be no harder than writing a blob of text.

SCIF accomplishes these goals by creating a metadata folder to serve each software module installed. A client that implements a controller for SCIF handles the mapping of a single entry point (a call to the executable “scif” in the examples above) to the respective installed applications.

## Modularity

A host with distinct, predictable locations for software modules and data is modular. The organization of these modules under a common root ensures that each carries a unique name. Further, while SCIF is not a package manager, this structure allows for easy movement of modules, if necessary. If a module carries with it complete information about installation and dependencies, it could easily be installed on another SCIF. The user does not need to look through mixed commands of a single build recipe (e.g., a Dockerfile or %post section in a Singularity recipe) and figure out which installation commands are associated with his software of interest.

### Kinds of Modularity

Modularity can be understood as the level of dimensionality that a user is instructed to operate, where the dimension might range from a single executable to multiple calls that form 1 step in a pipeline. For the purposes of this discussion, we again use an example that starts with a good reproducible practice: using a container to serve 1 or more scientific applications. For this scientific container, we suggest 3 kinds of modularity.

*Node.* For those familiar with container technology, it is commonly the case that an entire container is considered a module. An example is a container that performs the task of brain image registration. If the container itself is considered the module, the user would expect to provide an unregistered brain, call the container as an executable, and produce a registered brain as output. This container node would plug into higher-level orchestration tools that might include other neuroimaging preprocessing steps. This representation is ideal given that the container is expected to plug into a workflow manager and perform 1 task.

*Internal.* A second common scenario might be a single container that holds executables to perform different steps of a pipeline, perhaps so that the researcher can use the same container to run steps in serial or perform multiple steps in parallel. This container would come with multiple internal modules, each performing a series of commands for 1 step in the pipeline (e.g., for a container that performs variant calling, the step “mapping” might use internal commands from software bwa and samtools). The user doesn’t need to know the specifics of the steps but only how to call them. We call this level “internal modules” because without any formal structure for the contents of containers, they are hidden, internal executables that must be found or described manually.

*Development.* Containers can also serve modules that are represented at the ideal level for development. This means that the smallest units of software are exposed, such as the executables bwa and samtools. An improvement to this technology-oriented modularity would be to add a degree of scientific-oriented modularity, meaning that these units are specialized and stated to be intended for the scientific applications provided. It would be likely that a researcher developing a scientific pipeline would find this useful.

Given the different needs briefly explained above, it is clear that there is no correct level of dimensionality to define a module, but rather the level must be defined by the creator of the scientific container depending on its intended purpose. The definition of modularity then is variable and based on the needs of the creator and user. SCIF allows us to do this. We can define modules on the levels of single files or groups of software to perform a task. The metadata and organization of our preferences are automatically generated to create a complete and programmatically understandable software package.

## Solutions for Modularity

A discussion of modularity in the context of explaining the rationale for SCIF would not be complete without mentioning other solutions for achieving it. Several tools exist for managing environments including virtual environments [18], environment modules [19], and tools for automated configuration of systems [20,21]. While these tools are extensively used and adopted, SCIF is unique in that it exposes to the user an easy ability to define all interactions with a scientific application without needing extensive setup or command line knowledge. SCIF couples together features of defining not just an environment but also metadata, help documentation, entry points, tests, and files. In fact, SCIF can be used in conjunction with these tools to enhance them. For example, a SCIF on a shared cluster resource could have applications to manage loading sets of environment modules [19] paired with an entry point of interest and a help message for the user. A SCIF installed in a container in the context of a global comparison [22] would enhance the comparison by providing grouping of content. A SCIF on a local machine might be used to wrap common groupings of environments and commands and perform sanity checks before proceeding. SCIF, thus, can greatly enhance tools that already exist to manage modules, packages, and general environments. One of the soft goals of SCIF is be inclusive and work to enhance the many other good tools that already exist.

## Integrations and Tools

The following sections summarize how the SCIF fits nicely to allow for integrations, including but not limited to methods to generate reproducible containers, supporting tools for SCIF apps, workflow managers, and metrics for comparison.

### Container Bases

A scientist may choose to use containers to drive an analysis on a high-performance computing cluster or to deploy a container using a cloud provider service. For both of these use cases, we can imagine that the provider of the resource would want to develop an interface for the user to select software. It is often the case that a user has preference for a different version of software (e.g., for GPU, graphic processing units) to support an analysis or a different host operating system. By providing different software versions as SCIF apps with a selection of operating system bases, the provider can easily create such a tool. A hypothetical interface might use the following logic to guide the user’s choices and build a working container:

\begin{array}{l}
{\rm Operating\, System\, \hbox{--}\!>\, Library\, of\, Modules\, \hbox{--}\!> \,[user\, choice] }\\
\qquad\quad\hbox{--}\!>{\rm Container}
\end{array}

The user would ask for a selection of modules (software), and under the hood, the provider would choose the base image that best caters to the needs of the user. If the user has no preference for the operating system, the “Library of Modules” would instead be the first decision point:

\begin{array}{l}
{\rm Library\, of\, Modules\, \hbox{--}\!>\, [user\, choice]\, \hbox{--}\!> \,Operating\, System}\\
\qquad\quad{\rm  \hbox{--}\!> \,Container}
\end{array}

SCIF apps can easily plug into this kind of framework in that the “Library of Modules” is a listing of apps developed at the resource. Given shared organizational rules across bases afforded by SCIF, the only filter would be with regard to which software is suited for each operating system base, and this can be achieved by way of a label or derivation from a source container(s). In the case of a software module wanting to support multiple different hosts, the same rules would apply as they do now. Checks for the host architecture would come first to the installation procedure.

Under this framework, shared “base” containers can be generated for re-use, and despite a modular generation, the resulting containers are reproducible, and the internal organization of modules has a specific set of content that can be easily discovered for assessment. In fact, an even simpler setup could be achieved for a resource that didn’t want to use containers at all by simply providing a library of SCIF apps to interact with.

### Application Assessment

Assuming that a software or data module carries some kind of signature by way of its content or metadata, the next logical question is about the kinds of metrics that we can use for classification. Curation broadly encompasses the tasks of finding a scientific application that serves some function. Curation of a set of applications to derive structural and functional features can allow for representation of an entire host such as a container. Akin to the discussion on levels of modularity, we will start this discussion by reviewing the different ways that we might use to assess applications.

#### Manual Annotation

The obvious approach to curation is human-labeled organization, meaning that a person looks at a software package, calls it “biopython” in “python,” and then moves on. A user later might search for any of these terms and find the application. This same kind of curation might be slightly improved upon if it is done automatically based on the scientists domain of work (e.g., “biology”) or a journal the scientist has published in. We could even improve upon that by making associations of words in text where the application is defined or cited and collecting enough data to have some confidence of particular domains being associated [23]. Manual annotation might work well for small, manageable projects, and automated annotation might work given a large enough source of data to learn from, but overall this metric is unreliable. We cannot have certainty that every single container has enough citations to make automated methods possible or, in the case of manual annotation, that there is enough staffing to maintain it.

#### Functional Assessment

The second approach to assessing applications is functionally. We can view software as a black box that performs some task and assess the software based on comparing performance of that task. If 2 versions of a Python module produce the same output, despite subtle differences in the files (imagine the simplest case where the spacing is different), a functional assessment deems them identical. If we define a functional metric (e.g., timing how long it takes for a “Hello World” script to run implemented in different languages on the same host), we can rank languages from fastest to slowest or derive summary statistics. This kind of metric maps nicely to scientific disciplines for which the goal is to produce some knowledge about the world. However, understanding the reasons to explain the differences in performance is not possible if we don’t have a basic understanding of the application content. Given interaction with a container, in that containers are generally opaque, if we ask why the results of a functional assessment might be different, we cannot know.

Functional assessment also carries a nontrivial amount of work for the common scientist. Different domains would be required to robustly identify the most relevant metrics and data for this assessment. This kind of agreement is hard to come by. Thus, again we face a manual bottleneck that would minimally make functional assessment a slow process. This is not to say that functional assessment should not be done or is not important. It is paramount for scientists to understand the optimal way to achieve some specific goal, sometimes regardless of the costs. However, blind functional assessment does not reveal insights to content.

#### Application Assessment

An enhancement to functional assessment would be having the ability to associate different choices of software and protocol to the differences in outcomes that we see. For example, knowing the exact location of executables written in different programming languages to produce an output of “Hello World,” we can perform further comparisons on the contents or make the calls language agnostic by doing a trace of system calls [24]. For this kind of assessment to be possible, application organization and accessibility are paramount.

#### Competitive Assessment

As another example, imagine that a single scientific container provides 10 implementations of a sorting algorithm in Python. Running any one of the algorithms, each a SCIF app, would occur on the same input data and the same host. Given a predefined metric to assess each result, we might programatically parse over the Python scripts and compare imports and functions used across sorting algorithms. This is the idea of a Competitive assessment, which is a kind of functional assessment with 2 key components:

- A function and output of interest and
- A metric of goodness to assess the output.

The assessment is collaborative in that a scientist wanting to answer a specific scientific question would ask other scientists to write SCIF apps. Each contribution is then assessed by the predefined metric of goodness or assessed as the analysis code changes over time [25]. All of this might occur collaboratively with version control (e.g., Github [26]) linked to a Continuous Integration^3^. testing environment to run the contribution and make the assessment. SCIF would make this possible.

## Specification of SCIF

We now move into describing the specification itself (https://sci-f.github.io/spec). The Scientific Filesystem is a filesystem organization, a set of environment variables under the namespace SCIF (prefix “SCIF_”) and functions for interaction of the filesystem and variables. A client that implements SCIF must provide means to install the organizational structure and expose both the functions and environment variables. An integration that uses SCIF can expect the filesystem organization and environment variables. To give rationale for the development of SCIF, we start with a review of some basic background about Linux Filesystems.

### Traditional File Organization

File organization is likely to vary based on the host operating system, but arguably most Linux operating systems are similar to the Filesystem Hierarchy Standard (FHS) [7]. For this discussion, we disregard the inclusion of package managers, symbolic links, and custom structures and focus on the core of FHS. We discuss these locations in the context of how they do (or should) relate to scientific applications. It was an assessment of this current standard that led to the original development of SCIF.

#### Do Not Touch

Arguably, the following folders should not be touched by scientific software:

- /boot: boot loader, kernel files
- /bin: system-wide command binaries (essential for OS)
- /etc: host-wide configuration files
- /lib: system-level libraries
- /root: root’s home^4^
- /usr: read-only user applications
- /opt: package software applications
- /sbin: system-specific binaries
- /sys: system, devices, kernel features

While these locations likely have libraries and functions needed by the host to support software, it should not be the case that a scientist installs his or her software under any of these locations. The best contender here for scientific applications is /opt; however, it is missing specification for an organization, can be used for software that is not exclusive to a scientific application, and is likely to already be used by individuals or centers for a variety of software. While /usr could be a reasonable choice, it is typically used as a place to install other (nonscientific) applications and libraries, and it would be challenging to distinguish scientific applications from others. Further, the organization under /usr places standard directories (e.g., /bin, /lib, /etc, /share) on the first subfolder level and then allows for content from multiple applications in these subfolders. We could not have confidence about the content associated with each application, and in all of these locations, it would not be easy or intuitive to find scientific applications hidden with what is already provided by the host.

#### Variable and Working Locations

The following locations are considered working directories in that they hold variables defined at runtime or intermediate files that are expected to be purged at some point:

- /run: run time variables for running of programs
- /var: variables and logging
- /tmp: temporary location for users and programs
- /home: can be considered the user’s working space.^5^

#### Connections

Connections are devices and mount points. A host or scientific container will arguably always need to be able to support mount points that might be necessary from its host or resource, so it would be important for a scientific application to not be kept in these locations:

- /dev: essential devices
- /mnt: temporary mounts
- /srv: is for “site-specific data” served by the system. This might be a logical mount for cluster resources
- /proc: connections between processes and resources and hardware information

### SCIF File Organization

SCIF defines a single root base at /scif that includes 2 subfolder bases that can be known and consistently mounted across research clusters. The base of /scif/apps is where scientific applications reside, and /scif/data is where the corresponding data are found. These locations were chosen to be independent of any locations on traditional Linux filesystems for the sole purpose of avoiding conflicts. The SCIF takes the following structure, and we show the reader an example organization for a scientific application “foo”:

- /scif: the root of the entire SCIF
- /scif/apps: the root for scientific applications, where each subfolder is a single (modular) software application
- /scif/apps/foo: an example scientific application base, contains files relevant or needed
- /scif/apps/foo/bin: contains binaries for foo that should be on the $PATH
- /scif/apps/foo/lib: contains libraries for foo that should be on the $LD_LIBRARY_PATH
- /scif/apps/foo/scif: foo’s metadata folder for labels, environment, runscript, and help
- /scif/data: the root for scientific applications’ data, where each subfolder is intended for data belonging to a scientific application
- /scif/data/foo: the data folder for foo, where foo’s inputs and/or outputs can reliably be found

The above structure does not interfere with connections of its host or variable and working locations, and can be created by a SCIF client from a definition file.

#### Definition

A SCIF starts with a recipe to create it, which comes down to a text file that defines 1 or more scientific applications. The scif recipe is the primary method by which a user can define and then generate a SCIF. The recipe consists of sections, where the delineation of a section is determined by starting with a “%” and then being followed by a scientific application name that the section is relevant for.

\begin{array}{l}
\hbox{\% section foo}
\end{array}

Sections are defined for installation procedures, labels, help, environment, and defining entry points (Table 1).

**Table 1.** SCIF recipe sections

Section	Description
%appinstall	Commands executed relative to the application root to install the application. The writer of the recipe should expect the commands to be executed in $SCIF_APPROOT and thus write final outputs to $SCIF_APPBIN located at $SCIF_APPROOT/bin
%apphelp	Is written as a file called runscript.help in the application’s metadata folder where a client knows where to find it
%apprun	The “runscript” in the application’s metadata folder that is executed when the user asks to run the software
%applabels	Key value pairs to be written to a labels.json file in the application’s metadata folder
%appenv	Application-specific environment variables that are sourced when the application is active
%apptest	Tests specific to the application, with present working directory assumed to be the software module’s folder
%appfiles	A list of files to add from the host (or other location known to the integration or client) to the application root

Sections for each scientific application in the SCIF definition include commands to install dependencies, add files, and define environment variables, labels, and help for the application.

An example “hello-world” application definition is shown below, and these sections could appear in single text file along with other applications. In the example, a script “hello-world.sh” is written to the application’s root bin (see %appinstall), meaning that SCIF will add it automatically to the $PATH. The entry point of the application is to run this script (see %apprun) and environment variables active during interaction with the application (%appenv), along with labels (%applabels) and help (%apphelp). Not included in this recipe are sections to write tests (%apptest) or add files (%appfiles) so that the application can be installed from the file alone without any extra file dependencies.

\begin{array}{llll}
&{\rm \%\, apprun\, hello-world}     {\rm /bin/bash\, hello-world.sh}\\
&{\rm\%\, appinstall\, hello-world}\\
&\quad      {\rm echo\, ``echo'\, Hello\, World!'" >> \$SCIF_APPBIN/hello-world.sh}\\
&{\rm\%\, appenv\, hello-world}\\
 &\quad    {\rm chmod\, u+x\, \$SCIF_APPBIN/hello-world.sh}\\
 &\quad    {\rm THEBESTAPP=\$SCIF_APPNAME}\\
 &\quad   {\rm export\, THEBESTAPP}\\
&{\rm \% applabels\, hello-world} \\     
&{\rm MAINTAINER\, Vanessasaur}\\
&\quad     {\rm VERSION\, 1.0}\\
&{\rm \% apphelp}\\
&\quad {\rm This\, is\, an\, example\, ``Hello World"\, application.\, You\, can}\\ 
&\quad\quad\quad  {\rm install\, it\, to\, a} \\
 &\quad      {\rm Scientific\, Filesystem\, (scif)\, with\, the\, command:}\\
&\quad\quad     {\rm scif\, install\, hello-world.scif}\\
\\
&\quad  {\rm if\, you\, need\, to\, install\, scif:}\\
 &\quad  {\rm pip install scif}\\
\end{array}

The recipe is a text file with extension ‘.scif,’ and can serve as input to clients and integrations for SCIF. For example, a SCIF client would install a SCIF using a recipe “hello-world.scif” with the following command:

\begin{array}{l}
 {\rm   \$\, scif\, install\, hello-world.scif}
\end{array}

This is the complete set of steps for defining and creating a SCIF. For the interested reader, quick start tutorials are available (https://sci-f.github.io/tutorial-quick-start) along with complete code for the hello world example (https://github.com/sci-f/hello-world.scif) defined in the recipe above.

### Apps

Each installed scientific application has a subfolder under /scif/apps for its content and associated executables. Interaction with apps is driven by a predictable organization of file contents, paired with a core set of environment variables. Interaction between the 2 is driven by the control commands implemented by the client. In the following sections, we review the environment namespace and commands.

#### Environment Namespace

Discovery of data and scientific application folders is driven by way of environment variables. When the SCIF is interacted with in context of a specific scientific application:

\begin{array}{l}
{\rm \$\, scif\, run\, foo}
\end{array}

a set of environment variables about locations for the application’s data and executables is exposed. Variables is also exposed for these locations for other apps, and both sets of variables make it easy for the creator and user to reference locations without knowing the actual paths. Table **2** describes the environment variables that can be used in build recipes and are also available once the filesystem is built and interacted with. It includes variables to describe the entire SCIF (global) along with a specific app that is active. Present but inactive apps use the same namespace as the active app but are appended with an underscore and lowercase inactive app name (e.g., SCIF_APPENV_bar).

While SCIF is not a workflow manager, it follows naturally that the creator of a SCIF app might use these internal variables to have modules internally talk to 1 another. The user and creator do not need to know the structural specifics of the standard but only how to reference them. For example, a specific environment could be sourced by referencing SCIF_APPENV_bar and all defined environments discovered by way of searching for environment variables that start with SCIF_APPENV_*.

#### Functions

Interaction with a SCIF is driven by a set of functions (see “Commands” in https://sci-f.github.io/specification that are implemented by a client described in the specification. Briefly, we will demonstrate commands via interaction with the “hello-world” example shown previously.

*Applications.* List the scientific applications installed with a single command. Here, we discover a single application called “hello-world”:

\begin{array}{l}
{\rm \$\, scif\, apps}\\
{\rm SCIF\, [app]\quad\quad\quad              [root]}\\
{\rm 1\,  hello-world\, /scif/apps/hello-world}
\end{array}

*Help.* After we see a listing of scientific applications, we might want to ask for help for usage of the application.

\begin{array}{l}
{\rm \$\, scif\, help\, hello-world}\\
{\rm This\, is\, an\, example\, ``Hello\, World"\, application.\, You\, can\, install}\\ 
{\rm\qquad it\, to\, a\,  Scientific\, Filesystem\, (scif)\, with\, the\, command:}\\
{\rm\qquad scif\, install\, hello-world.scif.}
\end{array}

**Table 2.** SCIF environment namespace

Variable	Level	default or example	Description
SCIF_BASE	global	/scif	The root location for SCIF
SCIF_DATA	global	/scif/data	The root location for application data
SCIF_APPS	global	/scif/apps	The root location for installed apps
SCIF_SHELL	global	/bin/bash	Shell to use for “shell” commands
SCIF_PYSHELL	global	ipython	Python interpreter to use for pyshell command
SCIF_ENTRYPOINT	global	/bin/bash	The command to run given no runscript or app defined
SCIF_ENTRYFOLDER	global	SCIF_BASE	The entry folder to run the entry point command
SCIF_MESSAGELEVEL	global	INFO	A Client level of verbosity
SCIF_APPNAME	active	foo	The name of the active application
SCIF_APPDATA	active	/scif/data/foo	The data root for the active application
SCIF_APPROOT	active	/scif/apps/foo	The install root for the active application
SCIF_APPBIN	active	/scif/apps/foo/bin	The application bin that is automatically added to the path when active
SCIF_APPLIB	active	/scif/apps/foo/lib	The application lib that is automatically added to the path when active
SCIF_APPMETA	active	/scif/apps/foo/scif	The metadata folder
SCIF_APPHELP	active	/scif/apps/foo/runscript.help	A text file with help to print for the user to the terminal
SCIF_APPTEST	active	/scif/apps/foo/test.sh	A test script for the application
SCIF_APPRUN	active	/scif/apps/foo/runscript	The commands to run as the application entry point
SCIF_APPLABELS	active	/scif/apps/foo/scif/labels.json	A set of labels to describe the application
SCIF_APPENV	active	/scif/apps/foo/scif/environment.sh	An environment to be sourced for the application

SCIF provides an environment namespace that is available during the installation of the scientific application, along with its execution. Variables can describe an entire SCIF (global) or an active application (active). Present but not active application are represented with the active namespace, plus an underscore and lower application name (e.g., SCIF_APPENV_bar).

*Run.* Running a scientific application corresponds to executing the commands defined in the %apprun section:

\begin{array}{l}
{\rm \$\, scif\, run\, hello-world}\\
{\rm [hello-world]\, executing /bin/bash} \\
{\rm \quad /scif/apps/hello-world/scif/runscript}\\
 {\rm Hello\, World!}
\end{array}

*Exec.* Execute a command in context of the app “hello world.”

\begin{array}{l}
{\rm \$\, scif\, exec\, hello-world\, ls\, [e]SCIF_APPROOT}\\
{\rm [hello-world]\, executing /bin/ls \$SCIF_APPROOT}\\
{\rm bin}\\
{\rm lib}\\
{\rm scif}
\end{array}

Notice how we replace the typical $ for the environment variable with [e]. This is a strategy to ensure that environment variables do not get parsed on any host (and thus not passed to the scientific application).

*Inspect.* Inspection of a scientific application will return a data structure to see different sections defined for the application.

\begin{array}{l}
{\rm \$\, scif\, inspect\, hello-world}\\  
\{\\
{\rm \quad ``hello-world": \{}\\
{\rm \qquad ``apprun": [}\\
{\rm  \qquad\quad``/bin/bash\, hello-world.sh"}\\
\quad ],\\
{\rm \quad``appinstall": [}\\
{\rm \qquad ``echo\, "echo\, `Hello\, World!'`` >>}\\
 {\rm \qquad \$SCIF_APPBIN/hello-world.sh",}\\
{\rm \quad ``chmod u+x \$SCIF_APPBIN/hello-world.sh"}\\
\quad ],\\
{\rm \quad ``appenv": [}\\
{\rm \qquad ``THEBESTAPP=\$SCIF_APPNAME",}\\
{\rm \qquad``export\, THEBESTAPP"}\\
\quad],\\
{\rm \quad ``applabels": [}\\
{\rm \qquad ``MAINTAINER\, Vanessasaur",}\\
{\rm \qquad ``VERSION 1.0"}\\
\quad ],\\
{\rm \quad ``apphelp": [}\\
{\rm \qquad ``This\, is\, an\, example\, ``Hello\, World"\, application. You}\\
 {\rm \qquad\quad can\, install\, it\, to\, a",}\\
{\rm \qquad ``Scientific\, Filesystem\, (scif)\, with\, the\, command:",}\\
{\rm \qquad ``scif\, install\, hello-world.scif",}\\
{\rm  \qquad ``if\, you\, need\, to\, install\, scif:",}\\
{\rm \qquad ``pip\, install\, scif"}\\
\quad \quad]\\
\quad\}\\
\}
\end{array}

*Shell.* A shell allows the user to interact with the scientific application.

\begin{array}{l}
 \hbox{ \$ scif shell hello-world}\\
 \hbox{ [hello-world] executing /bin/bash}\\ 
 \hbox{ vanessa@vanessa-ThinkPad-T460s:/scif/apps/hello-world\$ env}\\
 \hbox{\qquad | grep SCIF\_}\\
 \hbox{ SCIF\_APPMETA=/scif/apps/hello-world/scif}\\
 \hbox{ SCIF\_DATA=/scif/data}\\
 \hbox{ SCIF\_APPHELP\_hello\_world=/scif/apps/hello-world/scif/runscript.}\\
 \hbox{\qquad help}\\
\end{array}

\begin{array}{l}
 \hbox{ SCIF\_APPDATA=/scif/data/hello-world}\\
 \hbox{ THEBESTAPP=\$SCIF\_APPNAME}\\
 \hbox{ SCIF\_APPENV=/scif/apps/hello-world/scif/environment.sh}\\
 \hbox{ SCIF\_APPROOT=/scif/apps/hello-world}\\
 \hbox{ SCIF\_APPRECIPE\_hello\_world=/scif/apps/hello-world/scif/hello-}\\
 \hbox{\qquad world.scif}\\
 \hbox{ SCIF\_APPDATA\_hello\_world=/scif/data/hello-world}\\
 \hbox{ SCIF\_APPLABELS=/scif/apps/hello-world/scif/labels.json}\\
 \hbox{ SCIF\_APPRUN=/scif/apps/hello-world/scif/runscript}\\
\hbox{ SCIF\_APPENV\_hello\_world=/scif/apps/hello-world/scif/}\\
 \hbox{\qquad environment.sh}\\
 \hbox{ SCIF\_APPRUN\_hello\_world=/scif/apps/hello-world/scif/runscript}\\
 \hbox{ SCIF\_APPLABELS\_hello\_world=/scif/apps/hello-world/scif/labels.}\\
 \hbox{ json}\\
 \hbox{ SCIF\_APPHELP=/scif/apps/hello-world/scif/runscript.help}\\
 \hbox{ SCIF\_APPLIB\_hello\_world=/scif/apps/hello-world/lib}\\
 \hbox{ SCIF\_APPS=/scif/apps}\\
 \hbox{ SCIF\_APPMETA\_hello\_world=/scif/apps/hello-world/scif}\\
 \hbox{ SCIF\_APPNAME\_hello\_world=hello-world}\\
 \hbox{ SCIF\_APPBIN\_hello\_world=/scif/apps/hello-world/bin}\\
 \hbox{ SCIF\_APPLIB=/scif/apps/hello-world/lib}\\
 \hbox{ SCIF\_APPRECIPE=/scif/apps/hello-world/scif/hello-world.scif}\\
 \hbox{ SCIF\_MESSAGELEVEL=INFO}\\
 \hbox{ SCIF\_APPBIN=/scif/apps/hello-world/bin}\\
 \hbox{ SCIF\_APPNAME=hello-world}\\
 \hbox{ SCIF\_APPROOT\_hello\_world=/scif/apps/hello-world}\\
\end{array}

In the example above, we look at the environment and see the full $SCIF_namespace. Notice that there are global variables for the SCIF (/SCIF_DATA and SCIF_APPS), along with general variables to always identify the active application (e.g., SCIF_APPROOT) along with application-specific variables that are active for all scientific applications installed (e.g., SCIF_APPROOT_hello_world).

*Pyshell.* For development, the referenced SCIF client exposes an interactive Python shell also for interaction with the SCIF.

\begin{array}{l}
\hbox{ \$ scif pyshell}\\
\hbox{ Found configurations for 1 scif apps}\\
 \hbox{ hello-world}\\
 \hbox{ [scif] /scif hello-world}\\
 \hbox{ Python 3.6.2 |Anaconda, Inc.| (default, Sep 22 2017, 02:03:08)}\\ 
 \hbox{ [GCC 7.2.0] on linux}\\
 \hbox{ Type ``help", ``copyright", ``credits" or ``license" for more}\\
 \hbox{\qquad information.}\\
 \hbox{ (InteractiveConsole)}\\
 \hbox{ >>>} 
\end{array}

### Data

SCIF does not enforce or state how the creator should use the data folders but rather encourages the creator to use the organization so that a user can intuitively know that any data for an application foo might go into /scif/data/foo and global data for the entire SCIF be in /scif/data. The latter would mean that another host could mount a host folder to /scif/data, and then generate results there. Beyond the top level folder specific to an application, the organization and format of data is up to the application to decide.^6^

#### Data Modularity

Akin to software modules, overlap in data modules is not allowed by way of the unique app names afforded by folders under a common directory. For example, let’s say we have an application called “foo.”

- Users and developers would know that foo’s data would be mounted or provided at /scif/data/foo.
- Importing of datasets within an app’s data folder that follow some other specific format [27] would be allowed, e.g., /scif/data/foo/bar1 and /sci/data/foo/bar2.
- An application’s data would be traceable to the application by way of its identifier. Thus, if I find /scif/data/foo, I would expect to find related software under /scif/apps/foo.

## Example Use Cases

SCIF is powerful in that it supports multiple general use cases for scientific and systems evaluation and high-level introspection. These use cases broadly fall in the areas of providing modular software, systems and metric evaluation, and guided collaboration to answer a scientific question. For all of these examples, installation of the SCIF in the container simply means installing the SCIF client and pointing it at a definition file.

\begin{array}{l}
\hbox {pip install scif}\\
 \hbox { scif install recipe.scif} 
\end{array}

This installation can happen on the host or in a container technology, and then the entry point to the container technology is the executable “scif” to provide the common interface to the SCIF. To demonstrate this, for the following example use cases, we will show the same commands executed side by side for each of 2 container technologies, Docker and Singularity, on the same host.

### Modular Software Evaluation

A common question pertains to evaluation of different solutions toward a common goal. An individual might ask “How does implementation A compare to implementation B as evaluated by 1 or more metrics?” For a systems administrator, the metric might pertain to running times, resource usage, or efficiency. A researcher might be interested in looking at variability (or consistency) of outputs. Importantly, it should be possible to give a SCIF serving such a purpose to a third party that does not know locations of executables, or environment variables to load, and the tests run equivalently. To ensure reproducibility, the tests might be distributed in a container. SCIF allows for this by way of providing modular software applications, each corresponding to custom environments, libraries, and potentially files.

#### Method

To demonstrate this use case, we developed a container that implements the most basic function for a program, a print to the console, for each of 17 languages (R, awk, bash, c, cat, clisp, cpp, csh, go, julia, octave, perl, python, ruby, rust, tcsh, zsh). The container is designed as a means to collect a series of metrics relative to timing and system resources for each language. The metrics pertain to system resources provided by the time [28] and strace [24] utilities. While it is necessary to define the applications installed at the point of creation, using SCIF means that a subsequent user can run the evaluation across modules (languages) without any previous knowledge with a simple for loop.

\begin{array}{l}
\hbox{ for app in \$(./hello-world apps)   \# Singularity}\\
\hbox{\qquad do}\\
 \hbox{\qquad ./hello-world run \$app}\\
 \hbox{ done}
\end{array}

The above example shows a Singularity image called “hello-world.” The general usage command is only slightly different with a Docker container.

\begin{array}{l}
\hbox{ docker run vanessa/hello-world.scif apps}\\
 \hbox{ docker run vanessa/hello-world.scif run bash}
\end{array}

#### Results

**Figure 1.**
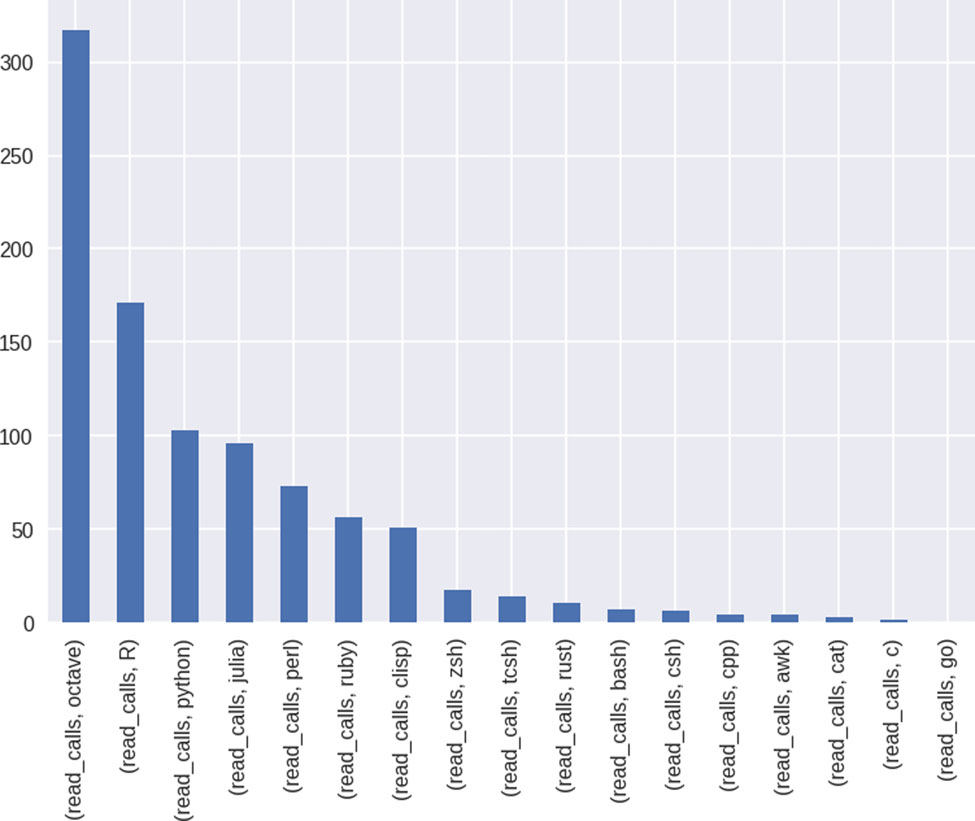
Assessing “read calls” across a range of different programming language implementations of “Hello World” shows a surprising range of differences and reflects common knowledge that more extensive programs (e.g., Octave) add complexity to the seemingly simple command.

To demonstrate the value of using SCIF in containers, we collected run time metrics for 19 SCIF apps, each of which printed “Hello World” using a different programming language. While it was necessary for the creator of the container to define the different language entry points, the analysis is able to run in entirety without knowing the languages. The resulting table of features pertaining to times and features demonstrates a wide span of differences between the seemingly identical calls. For example, Fig. 1 shows the differences in “read calls,” or the number of read commands to the filesystem issued when the simple “Hello World” command was run.

Closer inspection reveals facts about the programs that are common knowledge, such as shell programs having faster start up times than more substantial programs (e.g., octave, R, or Python). In fact, the basic differences between start times, reads and writes, and memory usage across this simple execution are surprising and give strong support for why scientific results can vary depending on the underlying architecture. Also, they give even stronger rationale for being able to assess the metadata about the software to reveal cause for the observed differences. Full results and additional analyses are available in this jupyter notebook, and a more detailed writeup is provided. The full analysis code and results are also provided.

### Diagnostic Metrics Evaluation

For this next use case, a scientist is interested in running a series of metrics over an analysis of interest. The scientist defines each metric as a SCIF application in a container build recipe alongside a primary entry point and builds a container called “metrics.” Each installed SCIF app can be thought of as a particular context to evoke an entry point of interest in the container, the container’s primary runscript, and the apps themselves are relatively agnostic to the entry point itself. Importantly, he can discover the metrics provided with the “scif apps” command

\begin{array}{l}
 \hbox{ \$ ./metrics}\\
 \hbox{ ``Hello World!"}\\
 \\
 \hbox{ \# Docker}\\
 \hbox{ \$ docker run vanessa/metrics.scif}\\
 \hbox{ ``Hello World!"}\\
\\
 \hbox{ \$ ./metrics apps}\\
 \hbox{\qquad custom}\\
 \hbox{\qquad linter}\\
 \hbox{\quad parallel}\\
 \hbox{\qquad strace}\\
 \hbox{\qquad\quad time}\\
 \hbox{ \# docker run vanessa/metrics.scif apps}
\end{array}

and then run a named metric easily by simply executing the container and specifying it.

\begin{array}{l}
\hbox{\$ ./metrics run time}\\
\hbox{\$ docker run vanessa/metrics.scif run time}
\end{array}

This particular container has several metrics to assess usage and timing of different resources (time), a complete trace of the call (strace), a static linter (linter), and a function to run the container’s runscript in parallel (parallel). Each of these SCIF apps serves as an example use case that is discussed in the following sections.

#### Metric example 1: evaluate software across different metrics

A system administrator or researcher concerned about evaluation of different software could add relevant metrics apps to the software containers and then easily evaluate each 1 with the equivalent command to the container. Importantly, since each evaluation metric is a modular application, the container still serves its intended purposes. As an example, here is a simple app to return a table of system traces for a specific app “main,” which is the primary runscript:

\begin{array}{l}
 \hbox{ \% apprun strace}\\
 \hbox{\quad exec strace -c -t scif run main}
\end{array}

With a simple check, the definition can be edited to run a system trace for a user-specified application:

\begin{array}{l}
\hbox{ \% apprun strace}\\
 \hbox{\qquad  if [ \$\# -eq 0 ]}\\
 \hbox{\qquad\quad  then}\\
 \hbox{\qquad\quad  exec strace -c -t scif run main}\\
 \hbox{\qquad  else}\\
 \hbox{\qquad\quad  exec strace -c -t scif run ``\$@"}\\
 \hbox{  fi}
\end{array}

The table returned assesses the runscript, and the interaction with the user looks like the following (truncated):

\begin{array}{l}
\hbox{\$ ./metrics run strace}\\
\hbox{[strace] executing /bin/bash /scif/apps/strace/scif/runscript}\\
\hbox{strace: -t has no effect with -c}\\
\hbox{[main] executing /bin/bash /scif/apps/main/scif/runscript}\\
 \hbox{ Hello World!}\\
\hbox{\% time\qquad   seconds\qquad usecs/call\qquad  calls\qquad errors syscall}\\
 \hbox{----------------}\quad \hbox{----------------}\quad \hbox{----------------}\quad \hbox{----------------}\quad \hbox{----------------}\\
\qquad\hbox{----------------}\\
\hbox{100.00\qquad\qquad    0.000008\qquad\qquad           0\qquad\qquad        40\qquad\qquad           munmap}\\
 \quad \hbox{0.00\qquad\qquad    0.000000\qquad\qquad           0\qquad\qquad       707\qquad\qquad           read}\\
 \quad\hbox{0.00\qquad\qquad    0.000000\qquad\qquad           0\qquad\qquad         1\qquad\qquad\quad           write}\\
 \quad\hbox{0.00\qquad\qquad    0.000000\qquad\qquad           0\qquad\qquad       426\qquad\qquad        42 open}\\
 \quad\hbox{0.00\qquad\qquad    0.000000\qquad \qquad          0\qquad\qquad       447\qquad \qquad          close}\\
\\
 \hbox {\# docker run --security-opt seccomp:unconfined}\\
 \hbox {\quad vanessa/metrics.scif run strace}\\
 \hbox { ...}
 \end{array}

Any user that adds the SCIF module to his or her container would immediately have this assessment for any SCIF application provided by his or her container. In the example below, we run a system trace for the SCIF application “custom.”

\begin{array}{l}
\hbox{\$ ./metrics run strace custom}\\
\hbox{\$ docker run --security-opt seccomp:unconfined}\\
\hbox{\quad vanessa/metrics.scif run strace custom}
\end{array}

The code and additional notes for this strace app are provided at https://www.github.com/sci-f/metrics.scif.

### Metric example 2: code quality and linting

A SCIF application can meet the needs to serve as a linter over a set of files. The example is provided here with a SCIF application “linter,” which runs a linter over a script.

\begin{array}{l}
\hbox{\$ ./metrics run linter}\\
\hbox{[linter] executing /bin/bash /scif/apps/linter/scif/runscript}\\
\hbox{No config file found, using default configuration}\\
\hbox{************* Module runscript}\\
\hbox{E:  1, 0: invalid syntax (<string>, line 1) (syntax-error)}\\
\\
\hbox{\# docker run vanessa/metrics.scif run linter}
\end{array}

This example lints a script defined by the SCIF creator (the SCIF executable itself), and the linter application could also accept the path to a custom file.

### Metric example 3: runtime evaluation

In that a metric can call another SCIF application, it could be easy to evaluate running a primary analysis under various conditions. As a simple proof of concept, here we are creating an application to execute another in parallel.

\begin{array}{l}
\hbox{\% apprun parallel}\\
 \hbox{ parallel /bin/bash ::: \$SCIF\_APPRUN\_main \$SCIF\_APPRUN\_main}\\
 \hbox{\quad \$SCIF\_APPRUN\_main}\\
\\
\hbox{\$ docker run vanessa/metrics.scif parallel}\\
\hbox{\$ ./metrics run parallel}\\
 \hbox{ [parallel] executing /bin/bash}\\
 \hbox{\quad /scif/apps/parallel/scif/runscript}\\
 \hbox{ Hello World!}\\
 \hbox{ Hello World!}\\
 \hbox{ Hello World!}
\end{array}

You might imagine a similar loop to run an analysis, modify a runtime or system variable for each loop, and save or print the output to the console.

### Contextual Running

It’s often common that a user will want to run an analysis in different environments. For example, a scientific analysis run locally would come down to executing the script, but run on a cluster would come down to submission of a job to a SLURM [29] or SGE [30] queue. To help distribute the analysis in different environments, a scientist could share a SCIF with an entry point for each use case. In the examples below, a container called “analysis” is an example of such a container.

\begin{array}{l}
\hbox{\$ ./analysis run slurm}\\
\hbox{\$ ./analysis run sge}\\
\hbox{\$ docker run vanessa/jobmaker.scif run slurm}\\
\hbox{\$ docker run vanessa/jobmaker.scif run sge}
\end{array}

The above commands pipe to the console a job configuration for each of slurm and sge that includes resources needed for the analysis. The resources (memory and time) were estimated during the build process and are carried forward with the scientific filesystem in the container, improving the ability to reproduce the work. This particular example for slurm and sge have been implemented.^7^

A cluster that provides containers for its users could provide submission scripts for optimal resource usage for each container.

### Interaction with Scientific Workflows

Interacting with scientific workflows is arguably the most common use case for scientific filesystems. A scientist is likely to use SCIF apps for 2 purposes:

- To provide development environments that expose software to test and develop pipelines
- To provide production applications to serve a final pipeline, ideally in a reproducible container to supplement a publication

SCIF can be of use to achieve both of these goals. For this example, we have implemented an equivalent pipeline using Singularity and SCIF for the CarrierSeq workflow [31], as well as adding SCIF to a previously created variant calling analysis that used Singularity and Docker^8^ Each of the 2 example containers provides modular access to the different software inside. By way of using SCIF, we have a lot of freedom in deciding on what level of functions we want to expose to the user. A developer will want easy access to the core tools (e.g., bwa, seqtk), while a user likely wants 1 level up, on the level of a collection of steps associated with some task (e.g., mapping).

#### Carrierseq Scientific Pipeline

For this example, we use a SCIF installed in a Singularity container [17] called “cseq” to perform a genomic analysis that includes a mapping algorithm, a statistical procedure (poisson), and a sorting procedure. We assume that an interested party has found the container “cseq”, has Singularity installed, and is curious about how to use it. The individual could first ask for help directly from the container.

\begin{array}{l}
\hbox{\$ ./cseq} \\
\hbox{[help] executing /bin/bash /scif/apps/help/scif/runscript}\\
\hbox{CarrierSeq is a sequence analysis workflow for low-input}\\
 \hbox{\quad nanopore}\\
\hbox{sequencing which employs a genomic carrier.}\\
\hbox{Github Contributors: Angel Mojarro (@amojarro),}\\
\hbox{\quad Srinivasa Aditya Bhattaru (@sbhattaru),}\\
\hbox{\quad Christopher E. Carr (@CarrCE),}\\
\hbox{\quad Vanessa Sochat (@vsoch).}\\
\hbox{fastq-filter from:}\\
 \hbox{\quad https://github.com/nanoporetech/fastq-filter}\\
\hbox{To see applications installed in the Scientific Filesystem:}\\
\hbox{scif apps}\\
\hbox{To run a typical pipeline, you might do:}\\
\hbox{scif run mapping}\\
\hbox{scif run poisson}\\
\hbox{scif run sorting}\\
\hbox{If you install in a container, the entry point should be}\\
 \hbox{\quad scif, and then}\\
\hbox{issue the above commands to it.}\\
\\
\hbox{\# Docker example}\\
\hbox{\$ docker run vanessa/cseq}
\end{array}

If we follow the instruction to list the SCIF apps, we find the container has an application that serves only to make the README.md easily accessible:

\begin{array}{l}
 \hbox{ \$ ./cseq apps}\\
 \hbox{\quad download}\\
 \hbox{\quad\quad help}\\
 \hbox{\quad mapping}\\
 \hbox{\quad poisson}\\
 \hbox{\quad readme}\\
 \hbox{ reference}\\
 \hbox{\quad sorting}\\
\hbox{\$ docker run vanessa/cseq apps}\\
\\
 \hbox{ \$ ./cseq help readme}\\
\hbox{\$ docker run vanessa/cseq help readme}\\
\\
 \hbox{ Print the repository's README.md to the console}\\
\\
 \hbox{ \$ ./cseq run readme}\\
 \hbox{ \$ docker run vanessa/cseq run readme}\\
\\
 \hbox{ \#\#\#\# CarrierSeq}\\
 \hbox{ \#\#\#\# About}\\
\\
 \hbox{ bioRxiv doi: https://doi.org/10.1101/175281}\\
\\
 \hbox{ CarrierSeq is a sequence analysis workflow for low-input}\\
\hbox{\quad nanopore}\\
 \hbox{\quad\quad sequencing which employs a genomic carrier.}\\
\\
\hbox{\quad  Github Contributors: Angel Mojarro}\\
 \hbox{\quad\quad\quad (@amojarro),}\\ 
\hbox{\quad\quad  Srinivasa Aditya Bhattaru (@sbhattaru),} \\
\hbox{\quad\quad  Christopher E. Carr (@CarrCE),} \\
\hbox{\quad\quad  and Vanessa Sochat (@vsoch).}\\
\\[-2pt]
 \hbox{ fastq-filter from: https://github.com/nanoporetech/fastq-filter}\\
\\[-2pt]
 \hbox{ [MORE]}
\end{array}

Metadata in the way of labels, environment, help, and the runscript and build recipes themselves are available for the whole container in either a json [32] or human readable format via an inspection command:

\begin{array}{l}
\hbox{\$ ./cseq inspect}\\
\hbox{\$ docker run vanessa/cseq inspect}
\end{array}

and also available on the level of individual apps:

\begin{array}{l}
\hbox{\$ cseq inspect mapping}\\
\hbox{\$ docker run vanessa/cseq inspect mapping}
\end{array}

The entire set of steps for running the pipeline provided by the container comes down to calling the different apps. Running the actual pipeline is outside of the scope of SCIF and can be solved by pipeline logic [3,33,34]. For this simple example, as an overall strategy, since the data are rather large, we are going to map a folder to the container’s data base where the analysis is to run. This directory, just like the modular applications, has a known and predictable location. The steps look like the following:

1. Download data to a host folder
2. For subsequent commands, map /scif/data to the host
3. Perform mapping step of pipeline
4. Perform poisson regression on filtered reads
5. Sort the results

The calls to the container to support this would be:

\begin{array}{l}
\hbox{\$ singularity run --bind data:/scif/data cseq run mapping}\\
\hbox{\$ singularity run --bind data:/scif/data cseq run poisson}\\
\hbox{\$ singularity run --bind data:/scif/data cseq run sorting}\\
\\
\hbox{\$ docker run -v \$PWD/data:/scif/data vanessa/cseq run mapping}\\
\hbox{\$ docker run -v \$PWD/data:/scif/data vanessa/cseq run poisson}\\
\hbox{\$ docker run -v \$PWD/data:/scif/data vanessa/cseq run sorting}
\end{array}

This would be enough to run the pipeline. What do the modules afford us? We can easily isolate metadata and contents related to each step or shell into the context to test:

\begin{array}{l}
\hbox{\$ ./cseq shell mapping}\\
\hbox{\$ docker run -it vanessa/cseq shell mapping}\\
\end{array}

We might also decide that we don’t like the “mapping” step and swap it out for 1 provided by a different container.

\begin{array}{l}
\hbox{\$ singularity run --bind data:/scif/data map-container run}\\
\hbox{\quad mapping}\\
\hbox{\$ singularity run --bind data:/scif/data cseq run poisson}\\
\\
\hbox{\$ docker run -v \$PWD/data:/scif/data vanessa/map-container}\\
\hbox{\quad run mapping}\\
\hbox{\$ docker run -v \$PWD/data:/scif/data vanessa/cseq run poisson}\\
\end{array}

A researcher who is incredibly interested in variations of 1 step (e.g., sorting) could provide an entire container just to serve those variations to then be used with carrierseq:

\begin{array}{l}
\hbox{\$ singularity run --bind data:/scif/data sort run quick}\\
\hbox{\$ singularity run --bind data:/scif/data sort run merge}\\
\\
\hbox{\$ docker run -v \$PWD/data:/scif/data vanessa/sort run quick}\\
\hbox{\$ docker run -v \$PWD/data:/scif/data vanessa/sort run merge}\\
\end{array}

Importantly, metadata and contents relevant to a specific step (e.g., “mapping”) are represented in the build recipe (the record of instructions that originally built the container and defined the apps) and the content of the filesystem itself (e.g., /scif/apps/mapping). The examples above show that SCIF provides a standard set of commands that could integrate easily into a workflow manager but also expose intuitive entry points for users that may not have expertise to use such a manager. The creator of the SCIF, the scientist, can carefully craft commands to be specific to his work, and the user is not expected to know the trivial details to use it. In fact, exposure to the details may even be a detriment if it confuses the user. This is a very different use case from a scientific developer’s, discussed next.

#### Carrierseq Development Container

The developer has a different use case — to have easy command line access to the lowest level of executables needed to develop an analysis pipeline. Given a global install of all software, without SCIF it would be necessary to look at $PATH to see what has been added to the path and then list executables in path locations to find new software installed to system locations like /usr/bin. The user can only assume that the creator of the host thought ahead to add these important executables to the path at all or has provided documentation for loading them. Unfortunately, there is no way to easily and programmatically “sniff” a filesystem to understand what changes were made and what tools are available. A development container created by developer Sam is likely not going to be understood by developer Stan. We would do well to create a development container installed with SCIF. For this discussion, we have created such a build recipe ^9^ and discuss usage of the SCIF in a container called cseq-dev.

For the CarrierSeq development container cseq-dev, instead of serving software on the level of the pipeline, we reveal the core software and tools that can be combined in specific ways to produce a pipeline step like “mapping.”

\begin{array}{l}
\hbox{\$ ./cseq-dev apps}\\
\hbox{bwa}\\
\hbox{fqtrim}\\
\hbox{help}\\
\hbox{python}\\
\hbox{seqtk}\\
\hbox{sra-toolkit}\\
\\[-2pt]
\hbox{\$ docker run vanessa/cseq:dev apps}
\end{array}

Each of the above apps can be used with commands “run”, “exec,” “inspect,” “shell,” or “test” to interact with the SCIF in context of a particular app. This means sourcing app-specific environment variables and adding executables associated with the app to the path. For example, I can use a simple app “python” to open the python interpreter in the container or shell into the container to test bwa:

\begin{array}{l}
\hbox{\#\#\#\#\# Open interactive python}\\
\hbox{\$ ./cseq-dev run python}\\
\hbox{\$ docker run -it vanessa/cseq:dev run python}\\
 \hbox{ [python] executing /bin/bash /scif/apps/python/scif/runscript}\\
 \hbox{ Python 2.7.9 (default, Jun 29 2016, 13:08:31)}\\ 
 \hbox{ [GCC 4.9.2] on linux2}\\
 \hbox{ Type ``help", ``copyright", ``credits" or ``license" for more}\\
 \hbox{\quad information.}\\
\hbox{  >>> }
\end{array}

\begin{array}{l}
\hbox{\#\#\#\#\# Load container with bwa on path}\\
\hbox{\#}\\
\hbox{\$ ./cseq-dev shell bwa}\\
\hbox{\$ docker run -it vanessa/cseq:dev shell bwa}\\
\hbox{[bwa] executing /bin/bash} \\
\hbox{\$ which bwa}\\
\hbox{\$ /scif/apps/bwa/bin/bwa}
\end{array}

These 2 CarrierSeq images that serve equivalent software, but enable very different use cases, are good examples of the flexibility of SCIF. The creator can choose the level of detail to expose to a user that doesn’t know how it was created. A lab that is using core tools for working with sequence data might have preference for the development container, while a finalized pipeline distributed with a publication would have preference for the first.

#### Singularity Scientific Example

Finally, we adopted an original analysis^10^ to compare Singularity vs. Docker on different cloud and local environments to give rationale for taking a SCIF apps approach over a traditional Singularity image. We compare the same pipeline implemented with SCIF and without SCIF as an example of how containers can provide the same software for equivalent functionality but, notably, have different organization that impacts discoverability. A detailed writeup of the rationale and use case is provided for the reader, along with the code base for the container^11^ In summary, without using SCIF, a filesystem with scientific applications is a black box. With SCIF, these same applications are available for discoverability and inspection.

### Research Evaluation

Containers aren’t only useful for running scientific pipelines, they are sources of information to discover good practices and features of scientific software. Having modular software apps allows for separation of files and executables for research from those that belong to the base system, enabling this kind of research. From a machine learning standpoint, the apps and corresponding metadata provide labels for a supervised algorithm to compare between scientific applications or even between the hosts where they are installed. In addition to the filesystem under /scif, the build recipe might also be parsed to see what software (possibly outside of the /scif root location) was intended to be shared between applications. Installation outside of the /scif root is meaningful in that it suggests global importance.

### Working Environments

SCIF has a very interesting use case when it comes to working environments, as each application can serve as an entry point to some custom context. Given that each application is associated with its own environment, labels, and executables on the $PATH, a SCIF can serve custom working environments, and this use case is very similar to modules [19]. Imagine that the execution of some command is not the goal of the container, but rather providing a set of environments around a software core. There is no minimum required set of sections to define an app, so a container that is intended as a “working container” might simply be a set of %appenv sections to define different named environments. Without any other section, the user is then able to interact with the custom, named environments. For example, we might have a container called “tensorflow” that can be shelled into with libraries for gpu on the path.

\begin{array}{l}
\hbox{\$ ./tensorflow shell gpu}
\end{array}

### Auditing and Logging

Although we do not delve into this use case, it should be noted that SCIF apps can provide logging and auditing for containers. A systems administration that builds and provides containers for his or her users might want to enforce running with a standard for logging and auditing [35]. Instead of asking the researcher to write this into his or her custom runscript, the snippet to perform the logging could be added as a SCIF app dynamically at build time and then the container run with this context.

For the complete specification, we direct the reader to the documentation base at https://sci-f.github.io.

## Community

To encourage sharing and distribution of useful apps, we have developed an online interface for easily exploring and sharing SCIF apps and generating recipes using the apps, available at https://sci-f.github.io/apps.

### Community Infrastructure

The interface is served from a Github repository that renders static template files into a complete website that includes search across all content, exploration by tag (e.g., language or operating system), and instruction by way of reading examples and tutorials. Programmatic access to all apps is provided with a RESTful API, as is a feed for interested users to be notified when new content is added. The interface also includes a recipe generator that allows a user to browse the site, save apps of interest in the browser’s local storage, and then combine them in a recipe file that can be downloaded in a compressed archive.

As an example integration, the Singularity container registry Singularity Hub^12^ provides a build service for the Singularity community and is designed to automatically extract complete metadata about apps that it discovers in containers. The metadata including app names, environments, and labels is indexed and search able on the Singularity Hub site. These tools, along with the ease of using SCIF, will greatly improve container transparency and recipe sharing.

### Contributing

Importantly, as the infrastructure is served from a Github repository, contributing does not require any expertise with web development or related technologies. The user can use Github to fork the repository, add a text file to a folder (_apps), and submit a pull request (PR) to evaluate the contribution. The text file itself has a header section that contains bullet pointed lists of metadata like name, tags, and files, and the remainder of the file is the sections for the SCIF application (e.g., %apprun hello-world). When the PR is approved, the contribution is automatically rendered into all areas of the community site. If an application includes associated files like scripts or configuration, these data are also easily added into a folder named equivalently to the file alongside it (e.g., _apps/hello-world/hello-world-bash.md would have associated files in _apps/hello-world/hello-world-bash). By way of using version control, all changes and contributions are tracked and credit allocated.

### Testing

Github also allows for complete testing of all contributions, and the repository is set up with a continuous integration (testing) service called CircleCI^13^ that checks the following:

- The file name for the application corresponds with the name declared in the file.
- The folder path under _apps also corresponds to the application’s file name. For example, an app located at _apps/hello-world/bash/ must start with hello-world-bash. Matching names to the folder structure ensures uniqueness of the names within the repository.
- The user has not provided any empty keys or values.
- Each declared file is included in the repository.
- The application minimally has a tag for 1 operating system^14^
- The header date is in valid format to be rendered correctly.
- Fields allowed in the header do not go beyond “author,” “title,” “date,” “files,” and “tags.”
- Required fields (“author,” “title,” “date,” and “tags”) are present.

Any contribution that does not meet these requirements will get feedback during the PR, and the contributor can address any issues. As soon as the content is merged into the master branch, it is immediately live on the site. The following are examples for the utility of this resource:

- A user can find useful examples and apps for his or her SCIF.
- A contributor can easily improve the SCIF specification or documentation via a PR.
- A user can contribute to a third-party software (e.g., Singularity) that has a native SCIF implementation.
- The user can contribute an application for others to use.
- A user can easily report an issue or ask a question to get help.

## Future Work

SCIF is exciting because it makes modular scientific application and container development and usage easier. It exists side-by-side with other solutions in the scientific software distribution ecosystem and is compatible with most, if not all, of them. The user can immediately inspect and see the software a SCIF provides and how to use it. The user can install additional software, use an application in a different container, or view metadata and help documentation. The developer is provided guidance for how and where to install and configure software, but complete freedom with regard to the software itself and the level of modularity to expose. The minimum requirements for any package are a unique name within the container and then any one of the needed sections. SCIF opens up an abstraction from underlying packaging logic and programming languages to work with scientific applications. This alone opens up new opportunities and great potential for other potential future use cases, discussed next.

### Mapping of Software Landscape

Given separation of the software from the host, we can more easily derive features that compare software modules. These features can be used with standard unsupervised clustering to better understand how groups of software are used together. We can further apply different labels to understand what modules are shared (or not shared) between scientific domains. We can find opportunity by discovering gaps (e.g., that a software module isn’t used for a particular domain) and then question why this is the case.

### Artificial Intelligence (AI) Generated Hosts

Given some functional goal, and given a SCIF that serves different variations of algorithms to achieve it alongside metrics about running the algorithms, we can (either by brute force or more elegantly) procedurally generate and optimize hosts for running the algorithms. An entire body of work can be made possible by way of installing SCIF applications to extract particular features or developing tools to externally interact with applications to evaluate features of the filesystem associated with different resource usage and resulting outcomes. In the long run, this kind of workflow offers promise to prune the host (e.g., container) landscape, and give insight to what it means to call a solution the “best.” The idea of procedure to build self-optimized hosts is, abstractly, a new kind of operating system that essentially designs itself [36].

## Discussion

In summary, SCIF is useful because it allows for:

- Flexible *modularity* where the definition of modularity is entirely based on the needs of the creator and user.
- *Reproducible practices* by way of providing portable environments with contents that are easily discovered.
- *Integration* with external tools.
- *Predictable structure* that distinguishes scientific content from the operating system base.
- *Community resources* including APIs, version control and testing, and open forums for tracking issues and discussions related to SCIF and SCIF apps.

This discussion would not be complete without a mention of limitations and suggested best practices.

### Limitations

It is important to distinguish the SCIF and the host that serves it. When a SCIF is installed to a container, it improves the discoverability of software for the container and thus greatly enhances the reproducibility of the container. While the container itself is portable and designed to contain all dependencies to support reproducibility, a SCIF module in and of itself is not guaranteed to be. For example, a user might define a module only with an %apprun section, implying that the folder only contains a runscript to execute. The user may have chosen to install dependencies for this script globally in the container because perhaps they are shared across multiple modules. Under these conditions, if another user expected to add the module to a different build recipe, the global dependencies would be needed too. The host operating system also needs to be taken into consideration. A module with dependencies installed from the package manager “yum” would not move seamlessly into a Debian base. However, appropriate checks and balances can be implemented to help with movement of applications between containers.

### Best Practices

#### Application Installation

To avoid missing dependencies, users are encouraged to include all dependency installs within the %appinstall section to make their applications maximally portable. It’s also good practice to use the %apptest section to ensure that an app that might have been newly installed is functioning as it should. Finally, metadata should be provided with apps about points of contact, usage and documentation, and supported operating system bases. To encourage this practice, we have added a test and requirements of specifying 1 or more operating systems for any module contributed at https://sci-f.github.io/apps.

#### Global vs Application Install

In the case of software that can be installed globally using a package manager, it is up to the creator to decide if a global vs. an application install is more appropriate. In practice, we have found that global installs tend to be larger, well maintained libraries (e.g., libraries installed with apt-get or package managers like pip), and having them installed globally to the container, to be shared among applications, is most appropriate. In the case of wanting multiple versions of the same software, an application install is most appropriate to keep the environments isolated. This decision is up to the generator of the SCIF.

Although we suggest using SCIF paired with Docker or Singularity for reproducibility, we do not enforce these particular technologies. SCIF can be extended to any general host or container technology that supports an install routine and has an execution entry point, and we encourage the community to foster discussion about this development.

## Conclusion

We have presented the SCIF and have shown examples of its functionality for interaction, development, and execution of scientific applications. This is an additional and complementary format to existing and successful approaches to scientific software distribution. The SCIF is advantageous in that its creator can embed his or her work with implied metadata about software and contents and generate an environment and organizational structure to support development and deployment. SCIF also makes it easier to package different entry points with a host and expose them easily to the user. However, this does not mean that the traditional approach of using a container as a general toolbox and distributing it with a series of external callers is bad or wrong. The community is provided with SCIF with a goal to support reproducible science and continued development of specialized filesystems. We hope that SCIF is useful for the larger community and encourage contributions to continue formalization of the format toward becoming a widely adopted standard.

## Abbreviations

API: Application Program Interface; APPS: Applications; BWA: Burrows-Wheeler Aligner, RRID:SCR_015853; HPC: High Performance Computing; SCIF: Scientific Filesystem, RRID:SCR_016105; SGE: Sun Grid Engine; SLURM: Simple Linux Utility for Resource Management; SEQTK: Sequence Processing Toolkit

## Resources

The following is a list of (possibly) related standards, formats and initiatives.

- File Hierarchy Standard
- Open Containers Initiative
- Common Workflow Language
- Fair Principles
- Open Standards
- https://reproducible-builds.org/
- DASPOS: https://daspos.crc.nd.edu
- TANGO: http://tango-project.eu/

## Code Availability

The Scientific Filesystem specification, client, and integrations are open source and freely available.

- Project name: Scientific Filesystem (SCIF)
- Project home page: https://sci-f.github.io
- Operating system(s): Linux
- Programming language: python,bash
- License: Apache AGPL

## Availability of supporting data

The software and code supporting the use cases in this article are available in several code repositories. Snapshots of the code are also available in the GigaDB repository [37].

- SCIF Client https://www.vsoch.github.com/scif/
- SCIF Documentation https://sci-f.github.io
- SCIF Apps and Resources https://sci-f.github.io/apps/

## Competing Interests

The authors declare that they have no competing interests.

## funding

V. S. is supported by the Stanford Research Computing Center and the Stanford School of Medicine.

## Author Contributions

V. S. conceptualized, implemented, developed, and tested the SCIF, along with the associated web applications, examples, and tutorials.

## Supplementary Material

GIGA-D-17-00289_Original_Submission.pdf

GIGA-D-17-00289_Revision_1.pdf

GIGA-D-17-00289_Revision_2.pdf

Response_to_Reviewer_Comments_Original_Submission.pdf

Response_to_Reviewer_Comments_Revision_1.pdf

Reviewer_1_Report_(Original_Submission) - Chris Richardson, PhD 20 Nov 2017 Reviewed

Reviewer_2_Report_(Original_Submission) -- Yaroslav Halchenko 12 Dec 2017 Reviewed

## Footnotes

1 A specification file to describe the steps to construct the container.

2
https://www.opencontainers.org/.

3
https://www.cirleci.com or https://www.travis-ci.org.

4 Unless you are using Docker, putting things here leads to trouble.

5 In that some container technologies (e.g., Singularity) mount the user’s home by default, and permissions in this location are user-specific, it is not advisable to assume stability in putting software here.

6 For example, when app “foo” is active, the environment variable SCIF_APPDATA references /scif/data/foo.

7
https://github.com/sci-f/jobmaker.scif.

8
https://github.com/vsoch/singularity-scientific-example.

9
https://github.com/vsoch/carrierseq

10
https://github.com/vsoch/singularity-scientific-example.

11
https://github.com/sci-f/scientific-example-ftw.

12
https://www.singularity-hub.org.

13
https://www.circleci.com.

14 to help determine compatibility.

## References

[1] Glatard T, Lewis LB, Ferreira da Silva R Reproducibility of neuroimaging analyses across operating systems. Front Neuroinform. 2015;9. 10.3389/fninf.2015.00012 PMC4408913 25964757

[2] Merkel D Docker: lightweight linux containers for consistent development and deployment. Linux J. 2014;2014.

[3] Docker-based solutions to reproducibility in science- Seven Bridges. 2015 https://blog.sbgenomics.com/docker-based-solutions-to-reproducibility-in-science/. Accessed: 17 Dec 2016.

[4] Hosny A, Vera-Licona P, Laubenbacher R AlgoRun: a Docker-based packaging system for platform-agnostic implemented algorithms. Bioinformatics. 2016;32:2396–98. 27153722 10.1093/bioinformatics/btw120 PMC6280798

[5] Moreews F, Sallou O, Ménager H BioShaDock: a community driven bioinformatics shared Docker-based tools registry. F1000Res. 2015;4:1443. 26913191 10.12688/f1000research.7536.1 PMC4743153

[6] Boettiger C An introduction to Docker for reproducible research, with examples from the R environment. 2014.

[7] Linux Filesystem Hierarchy.

[8] Wikipedia contributors. Comparison of file systems. 2016 https://en.wikipedia.org/w/index.php?title=Comparison_of_file_systems&oldid=751048657. Accessed: 23 Nov 2016.

[9] Overview of Docker Compose. https://docs.docker.com/compose/. Accessed: 9 Jan 2016.

[10] Di Tommaso P, Palumbo E, Chatzou M The impact of Docker containers on the performance of genomic pipelines. PeerJ. 2015;3:e1273. 26421241 10.7717/peerj.1273 PMC4586803

[11] Curcin V, Ghanem M Scientific workflow systems - can 1 size fit all? In: 2008 Cairo International Biomedical Engineering Conference; 2008 p. 1–9.

[12] [PDF]A Survey of Data-Intensive Scientific Workflow Management - Inria.

[13] Conda— Conda documentation. Accessed: 2018-2-6. https://conda.io/docs/. Accessed: 6 Feb 2018.

[14] Hoste K Installing software for scientists on a multi-user HPC system.

[15] Kaczmarzyk J neurodocker.

[16] Chirigati F, Rampin E, Shasha D ReproZip: Computational Reproducibility With Ease.

[17] Kurtzer GM, Sochat V, Bauer MW Singularity: Scientific Containers for Mobility of Compute. 10.1371/journal.pone.0177459 PMC5426675 28494014

[18] Smith JE, Nair R Virtual Machines: Versatile Platforms for Systems and Processes. The Morgan Kaufmann Series in Computer Architecture and Design Series. Morgan Kaufmann Publishers; 2005.

[19] Furlani JL, Osel PW Abstract Yourself With Modules. In: Proceedings of the 10th USENIX Conference on System Administration. LISA ’96. Berkeley, CA, USA: USENIX Association; 1996 p. 193–204.

[20] Warewulf http://warewulf.lbl.gov/trac. Accessed: 5 Dec 2016.

[21] Wikipedia contributors. List of build automation software; 2016 https://en.wikipedia.org/w/index.php?title=List_of_build_automation_software&oldid=745372506. Accessed: 23 Nov 2016.

[22] container-diff.

[23] Yarkoni T, Poldrack RA, Nichols TE Large-scale automated synthesis of human functional neuroimaging data. Nat Methods. 2011;8:665–70. 21706013 10.1038/nmeth.1635 PMC3146590

[24] strace(1): trace system calls/signals - Linux man page. https://linux.die.net/man/1/strace. Accessed: 11 Sep 2017.

[25] Beaulieu-Jones BK, Greene CS Reproducible Computational Workflows with Continuous Analysis; 2016. 10.1038/nbt.3780 PMC6103790 28288103

[26] Understanding the GitHub Flow. https://guides.github.com/introduction/flow/. Accessed: 26 Jan 2017.

[27] Gorgolewski KJ, Auer T, Calhoun VD The brain imaging data structure, a format for organizing and describing outputs of neuroimaging experiments. Sci Data. 2016;3:160044. 27326542 10.1038/sdata.2016.44 PMC4978148

[28] time(1)- Linux manual page. http://man7.org/linux/man-pages/man1/time.1.html. Accessed: 26 Jan 2017.

[29] Slurm Workload Manager. Accessed: 2016-12-6. https://slurm.schedmd.com/plugins.html.

[30] SGE Manual Pages http://gridscheduler.sourceforge.net/htmlman/manuals.html. Accessed: 4 Nov 2015.

[31] Mojarro A, Hachey J, Ruvkun G, Zuber MT, Carr CE CarrierSeq: a sequence analysis workflow for low-input nanopore sequencing; 2017. 10.1186/s12859-018-2124-3 PMC5872496 29587645

[32] contributors W JSON; 2015 https://en.wikipedia.org/w/index.php?title=JSON&oldid=692109528. Accessed: 24 Nov 2015.

[33] Köster J, Rahmann S Snakemake–a scalable bioinformatics workflow engine. Bioinformatics. 2012;28:2520–22. 22908215 10.1093/bioinformatics/bts480

[34] Data Sciences Platform @ Broad Institute. WDL | Home. https://software.broadinstitute.org/wdl/. Accessed: 6-Feb-2018.

[35] Stodden V Reproducibility in Computational and Experimental Mathematics.

[36] Myers K At the Boundary of Workflows and AI. AAAI Technical Report.

[37] Sochat V Supporting data for “The Scientific Filesystem (SCIF); 2018. GigaScience Database. http://dx.doi.org/10.5524/100420. 10.1093/gigascience/giy023 PMC5952957 29718213

