# Supplementary material for: The Scientific Filesystem
Source: Gigascience. 2018 Mar 13;7(5):giy023. doi: 10.1093/gigascience/giy023 (PMC5952957; doi:10.1093/gigascience/giy023)
Supplement: GIGA-D-17-00289_Original_Submission.pdf [file giy023_giga-d-17-00289_original_submission.pdf]

GigaScience

The Standard Container Integration Format (SCI-F)

--Manuscript Draft--

**Manuscript Number:**	GIGA-D-17-00289
**Full Title:**	The Standard Container Integration Format (SCI-F)
**Article Type:**	Technical Note
**Funding Information:**	
**Abstract:**	Background, Here we present the Standard Container Integration Format (SCI-F), an organizational format for internally modular scientific containers that makes development and usage easier. With SCI-F, a single, reproducible container to deploy a published scientific workflow can have multiple exposed entry points each that includes its own environment, metadata, installation steps, tests, files, and a primary executable script. We will start by reviewing the background and rationale for a container organizational format, and how SCI-F achieves the goals of modularity, transparency, parsability, and consistency. We then review the organizational structure of the standard, and the different levels of internal modules ("apps") that it affords. Finally, we demonstrate that SCI-F is useful by implementing and discussing several use cases, and releasing SCI-F with Singularity software version 2.4. Results, We use SCI-F to evaluate container software, provide metrics, serve scientific workflows, and execute a primary function under different contexts. To encourage collaboration and sharing of apps, we have developed an open source, version controlled, tested, and programmatically accessible web infrastructure at https://containers-ftw.github.io/apps. The ease of using SCI-F to develop scientific containers offers promise for scientists to easily generate self-documenting containers that are programmatically parseable, exposing software and associated metadata, environments, and files to be quickly found and used.
**Corresponding Author:**	Vanessa Sochat, PhD<br>Stanford University School of Medicine<br>Stanford, CA UNITED STATES
**Corresponding Author Secondary Information:**	
**Corresponding Author's Institution:**	Stanford University School of Medicine
**Corresponding Author's Secondary Institution:**	
**First Author:**	Vanessa Sochat, PhD
**First Author Secondary Information:**	
**Order of Authors:**	Vanessa Sochat, PhD
**Order of Authors Secondary Information:**	
**Opposed Reviewers:**	
**Additional Information:**	
**Question**	**Response**
Are you submitting this manuscript to a special series or article collection?	No
**Experimental design and statistics**<br><br>Full details of the experimental design and statistical methods used should be given in the Methods section, as detailed in our Minimum Standards Reporting Checklist. Information essential to interpreting the	Yes

Powered by Editorial Manager® and ProduXion Manager® from Aries Systems Corporation

data presented should be made available in the figure legends.<br><br>Have you included all the information requested in your manuscript?	
**Resources**<br><br>A description of all resources used, including antibodies, cell lines, animals and software tools, with enough information to allow them to be uniquely identified, should be included in the Methods section. Authors are strongly encouraged to cite Research Resource Identifiers (RRIDs) for antibodies, model organisms and tools, where possible.<br><br>Have you included the information requested as detailed in our Minimum Standards Reporting Checklist?	Yes
**Availability of data and materials**<br><br>All datasets and code on which the conclusions of the paper rely must be either included in your submission or deposited in publicly available repositories (where available and ethically appropriate), referencing such data using a unique identifier in the references and in the "Availability of Data and Materials" section of your manuscript.<br><br>Have you have met the above requirement as detailed in our Minimum Standards Reporting Checklist?	Yes

Powered by Editorial Manager® and ProduXion Manager® from Aries Systems Corporation

Manuscript

Click here to download Manuscript standard-container-integration.pdf

OXFORD (GIGA)n SCIENCE

GigaScience, 2017, 1–14

doi: xx.xxxx/xxxx
Manuscript in Preparation
Paper

PAPER

The Standard Container Integration Format (SCI-F)

Vanessa Sochat[1,*,†]

[1]Stanford Research Computing Center and [2]Stanford University School of Medicine

*vsochat@stanford.edu

Abstract

Background, Here we present the Standard Container Integration Format (SCI-F), an organizational format for internally modular scientific containers that makes development and usage easier. With SCI-F, a single, reproducible container to deploy a published scientific workflow can have multiple exposed entry points each that includes its own environment, metadata, installation steps, tests, files, and a primary executable script. We will start by reviewing the background and rationale for a container organizational format, and how SCI-F achieves the goals of modularity, transparency, parsability, and consistency. We then review the organizational structure of the standard, and the different levels of internal modules ("apps") that it affords. Finally, we demonstrate that SCI-F is useful by implementing and discussing several use cases, and releasing SCI-F with Singularity software version 2.4. **Results**, We use SCI-F to evaluate container software, provide metrics, serve scientific workflows, and execute a primary function under different contexts. To encourage collaboration and sharing of apps, we have developed an open source, version controlled, tested, and programmatically accessible web infrastructure at https://containers-ftw.github.io/apps. The ease of using SCI-F to develop scientific containers offers promise for scientists to easily generate self-documenting containers that are programmatically parseable, exposing software and associated metadata, environments, and files to be quickly found and used.

Key words: containers; reproducibility; singularity; hpc; workflows; linux containers; standard

Introduction

For quite some time, our unit of understanding computer systems has been based on the operating system. It is the level of magnification at which we operate (personal computers) and the starting point for working with data, software, and services [1]. With the rising popularity of Linux containers [2, 3, 4, 5], we have carried forward that unit of operation to be the primary content of containers. While this practice affords reproducibility by way of providing encapsulated, portable environments, in the same way that we cannot reliably predict how to call a program on a colleague's computer without having outside knowledge, the contents of the containers are not internally modular or programmatically understandable. These containers, without special knowledge from the creator, are akin to black boxes. For the scientific community, this is a barrier to providing scientific containers as reproducible products.

How might we interact with these black box containers? In the best case scenario, executing the container will execute some main driver of the software with instructions for usage, however this model is not well suited if there is more than one executable. An assessment of a container's ability to fully communicate its usage can start with a series of simple questions. Can the executables for the analysis be predictably found? If a particular directory is bound from the host, is any important container content lost? Can we untangle the scientist's contributions from the base operating system? Without a standardized approach to ensure internal consistency and modularity, these questions are not easily answerable. A standard is needed to make it easy for the creator to define multiple applications within a single container, and for those applications to expose themselves throughout the life-cycle of the container [6]. Such a standard must address the following issues:

- Containers are not **consistent** to allow for comparison across containers generated by different individuals.
- Containers are not **transparent**. If I discover a container and do not have any prior knowledge or metadata, a known function may be completely concealed.
- Container contents are not programmatically **understand-**

Compiled on: October 30, 2017.
Draft manuscript prepared by the author.

Key Points

- Container internal software, environment, and metadata are not easily exposed.
- Container inspection and predictability is essential for scientific reproducibility
- SCI-F makes it easy to generate modular, predictable, and programmatically understandable containers.

able. I should be able to inspect a container, know exactly the functions available to me, ask for help for a function, or how and where to interact with inputs and outputs.

- Container internal infrastructure is not **modular**. We would be weary to export an entire container into another because of overlapping content.

The basis of these core problems can be reduced to the fact that we are being forced to operate on a level that no longer makes sense given the problem at hand. Furthering reproducible practices calls for optimized definition and modular organization of applications and data, and this is a different set of goals than structuring one system per the Filesystem Hierarchy Standard [7, 8]. This goal is also not met by assigning one software package per container, because there is huge redundancy with regard to the duplicated filesystem, and the added software is now hiding amongst it.

The above problems also hint that the generation of containers is not easy. When a scientist starts to write a build specification [1], he probably doesn't know where to install software, or perhaps that a help file should exist, or that metadata about the software should be served by the container. To generate containers that easily integrate with external hosts, data, and other containers, the generation software needs to capture this content automatically.

Based on these problems, it is clear that we need direction and guidance on how to organize multiple applications and data within a single container in a way that affords modularity, programmatic accessibility, transparency, and consistency. This document will review the rationale and use cases for the Standard Container Integration Format (SCI-F). We will first review the goals of the architecture, followed by integrations and tools, and then they organizational standard itself. To demonstrate utility, we describe several implemented use cases for assessment of container metrics and running scientific workflows. The document that describes the specification for SCI-F is openly available for critique and contribution at https://containers-ftw.github.io/SCI-F/, and contributions from the community are encouraged.

Goals

The Standard Container Integration Format (SCI-F) establishes an overall goal to make containers consistent, transparent, parseable, and internally modular. Using Singularity [9], we start with a reproducible encapsulated environment, and SCI-F further enhances this base. We assert that for a container to conform to SCI-F, it must:

- Be **consistent** to allow for comparison. I am able to easily discover relevant software and data for one or more applications defined by the container creator.
- Be **transparent**. If I discover a container and do not have any prior knowledge or metadata, the important executables and metadata are revealed to me.
- Make container contents easily available for introspection, meaning the container is programmatically **parseable**. I can run a function over a container, and know exactly the executables and options available to me.
- Provide container internal infrastructure that is **modular**. Given a set of SCI-F apps from different sources, I can import different install routines and have assurance that environment variables defined for each are sourced correctly for each, and that associated content does not overwrite previous content. Each software and data module must carry, minimally, a unique name and install location in the system.

To be clear, this is not a specification for a container image [2], or a workflow using containers [10, 11, 12, 13]. Although these goals match nicely with efforts for workflow and image standardization, SCI-F is a specification for modular organization of content within the image, untangled from the file system and image itself. Any container technology that implements SCI-F to achieve the goals of consistency, transparency, and modularity will provide an easy means to adopt this internal structure and expose it for use. Each of the specific goals in context of the assertions is discussed in more detail in the following sections. For these goals, we introduce the idea of container "apps," or subfolders corresponding to an internal module that conform to a predictable internal organization under /scif/apps.

Consistency

Given the case of two containers with the same software installed, it should be the case that the software with some unique resource identifier (commonly a name and version) and any included data can be consistently found. To achieve this goal, SCI-F defines a new root folder, /scif, a name that should have minimal conflict with existing cluster resources. Under this folder are separate folders for each of software modules, "container apps," under /scif/apps, and data under /scif/data. Under these two folders is where the container generation software generates subfolders for internally modular installed applications (apps). For example, a container with applications foo and bar would have them installed as follows:

```
/scif
    /apps
        /bar
        /foo
```

If two containers both have foo installed, we would know to find the installation under /scif/apps/foo. Data takes a similar approach. We define a new root folder, /scif/data, with a similar subfolder organization:

```
/scif
    /data
```

1 a specification file to describe the steps to construct the container

2 https://www.opencontainers.org/

```
    /bar
    /foo
```

A container in a workflow that knows to execute the foo application would also give the user guidance about where to mount to write output, or find inputs, and these locations would not conflict with another app. This exposure of content to support integration with workflow managers is the rationale for "Integration" in the organizational format's name. Although SCI-F is not a workflow specification or manager, the exposure of consistent data and executable locations makes it a powerful tool when paired with one.

Transparency

Arguably, when we want to know about a container's intended use, we don't care so much about what the underlying operating system is. We would want to subtract this base first, and then glimpse at what remains. Given the consistent organization above, we can easily determine the container's interesting [3] software with a simple command to list apps:

```
$ singularity apps containers.simg

  bar
  foo
```

We can predictably find and investigate a particular software given that we now know the name. In the example below, we demonstrate that a container used in the context of a particular app (foo), exposes important information via environment variables.

```
$ singularity shell --app foo container.simg

Singularity: Invoking an interactive shell within container...
$ echo $SINGULARITY_APPNAME
foo

$ echo $SINGULARITY_APPROOT
/scif/apps/foo

ls $SINGULARITY_APPDATA
input  output
```

The app can also be run, asking a container to call its defined executable.

```
$ singularity run --app foo container.simg
RUNNING FOO
```

The uniqueness of the base path /scif is important because of mounting. For hosts that do not support overlayfs [9], the mount point must also exist on the host, and so the primary folders for The Standard Container Integration Format should not interfere with any that exist on the host. (e.g., /opt). From a high level, we are introducing a simply formatted addition to the standard Linux File System Hierarchy, a folder at the root that starts with /scif that makes an assertion that "the content under this base is exclusively relevant to the purpose of the container, and not the base operating system."

3 interesting to the user means a primary softwre module that is relevant to the container's intended purpose

Parsability

Parsability comes down to programmatic accessibility. This means that, for each software module installed, we need to be able to do the following:

- **provide metadata** A software module might have a version, a link to further documentation, an author list, or other important metadata values that should be programmatically accessible.
- **provide an entry point** Different software modules within a container should each be able to define an entry point [4].
- **provide help** Given an entry point, or if a user wants to understand an installed application, it should be the case that a command can be issued to view documentation provided for the software. For the developer, adding this functionality should be no harder than writing a blob of text.

SCI-F accomplishes these goals by creating a metadata folder to serve each software module installed within the container. In the case of the Singularity implementation, this metadata folder mirrors the container's global metadata folder [9].

Modularity

A container with distinct, predictable locations for software modules and data is modular. The organization of these modules under a common root ensures that each carries a unique name. Further, this structure allows for easy movement of modules between containers. If a module carries with it complete information about installation and dependencies, it could easily be installed in another container. The user does not need to look through mixed commands of a single build recipe (e.g., a Dockerfile or %post section in a Singularity recipe) and figure out which installation commands are associated with his software of interest.

Kinds of Modularity

Modularity can be understood as the level of dimensionality that a user is instructed to operate, where the dimension might range from a single executable to multiple calls that form one step in a pipeline. For the purposes of this discussion we suggest three different kinds.

Node. For those familiar with container technology, it is commonly the case that an entire container is considered a module. An example is a container that performs the task of brain image registration. If the container itself is considered the module, the user would expect to provide an unregistered brain, call the container as an executable, and produce a registered brain as output. This container node would plug into higher level orchestration tools that might include other neuroimaging preprocessing steps. This representation is ideal given that the container is expected to plug into a workflow manager and perform one task.

Internal. A second common scenario might be a single container that holds executables to perform different steps of a pipeline, perhaps so that the researcher can use the same container to run steps in serial, or perform multiple steps in parallel. This container would come with multiple internal modules, each performing a series of commands for one step in the

4 An entry point is a function or script to call when a container is used as an executable. The current "runscript" for a Singularity container, or the Dockerfile ENTRYPOINT and CMD are examples

pipeline (e.g., for a container that performs variant calling, the step “mapping” might use internal commands from software bwa and samtools). The user doesn’t need to know the specifics of the steps, but how to call them. We call this level “internal modules” because without any formal structure for the contents of containers, they are hidden, internal executables that must be found or described manually.

Development. Containers can also serve modules that are represented at the ideal level for development. This means that the smallest units of software are exposed, such as the executables bwa and samtools. It would be likely that a researcher developing a scientific pipeline would find this useful.

Given the different needs briefly explained above, it is clear that there is no correct level of dimensionality to define a module, but rather the level must be defined by the creator of the container depending on its intended purpose. The definition of modularity, then is variable, and based on the needs of the creator and user.

What is needed is an ability for the creator of a container to implicitly define this level of usage simply by way of creating the container. SCI-F allows us to do this. We can define modules on the levels of single files, or groups of software to perform a task. The metadata and organization of our preferences is automatically generated to create a complete, and programmatically understandable software package.

Integrations and Tools

The following sections summarize how the Standard Container Integration Format fits nicely to allow for integrations, including but not limited to methods to generate reproducible containers, supporting tools for SCI-F apps, workflow managers that use containers, and metrics for comparison.

Container Bases

It is often the case that a user has preference for a different version of software (e.g., for GPU, graphic processing units) to support an analysis. By providing different software versions as modules, and containers with base operating systems to install the modules, SCI-F allows for provision of interactive tools for users to “choose your base” and then “add your software.” A hypothetical interface might use the following logic to guide the user’s choices, and build a working container:

```
Operating System --> Library of Modules --> [user choice] -->
      Container
```

The user would ask for a selection of modules (software), and under the hood the provider would choose the base image that best caters to the needs of the user. If the user has no preference for the operating system, the "Library of Modules" would instead be the first decision point:

```
Library of Modules --> [user choice] --> Operating System -->
      Container
```

SCI-F apps can easily plug into this kind of framework in that the "Library of Modules" is a listing of apps developed at the resource. Given shared organizational rules across bases afforded by SCI-F, the only filter would be with regard to which software is suited for each base, and this can be achieved by way of a label or derivation from a source container(s). In the case of a software module wanting to support multiple different hosts, the same rules would apply as they do now. Checks for the host architecture would come first to the installation procedure.

Under this framework, shared "base" containers can be generated for re-use, and despite a modular generation, the resulting containers are reproducible, and the internal organization of modules has a specific set of content that can be easily found for container assessment.

Container Assessment

Assuming that a software or data module carries some kind of signature by way of its content or metadata, the next logical question is about the kinds of metrics that we can use for classification. Container curation broadly encompasses the tasks of finding a container that serves some function, or representing containers by way of structural or functional features that can be easily compared. Akin to the discussion on levels of modularity, we will start this discussion by reviewing the different ways that we might use to assess containers.

Manual Annotation

The obvious approach to container curation is human labeled organization, meaning that a person looks at a software package, calls it “biopython” in “python” and then moves on. A user later might search for any of these terms and find the container. This same kind of curation might be slightly improved upon if it is done automatically based on the scientists domain of work (e.g. “biology”), or a journal published in. We could even improve upon that by making associations of words in text where the container is defined or cited, and collecting enough data to have some confidence of particular domains being associated [14]. Manual annotation might work well for small, manageable projects, and automated annotation might work given a large enough source of data to learn from, but overall this metric is unreliable. We cannot have certainty that every single container has enough citations to make automated methods possible, or in the case of manual annotation, that there is enough manpower to maintain it.

Functional Assessment

The second approach to assessing containers is functionally. We can view software as a black box that performs some task, and assess the software based on comparing performance of that task. If two different version of a Python module produce the same output, despite subtle differences in the files (imagine the simplest case where the spacing is different) a functional assessment deems them identical. If we define a functional metric (e.g. timing how long it takes for a "Hello World" script to run implemented in different languages), we can rank languages from fastest to slowest, or derive summary statistics. This kind of metric maps nicely to scientific disciplines for which the goal is to produce some knowledge about the world. However, understanding the reasons to explain the differences in performance is not possible if we don’t have a basic understanding of the container content. When containers are internally opaque, if we ask why the results of a functional assessment might be different, we cannot know.

Functional assessment also carries a non-trivial amount of work for the common scientist. Different domains would be required to robustly identify the metrics, data, and most relevant for this assessment, This kind of agreement is hard to come by. Thus, again we face a manual bottleneck that would minimally make functional assessment a slow process. This is not to say that functional assessment should not be done or is not important. It is paramount for scientists to understand the optimal way to achieve some specific goal, sometimes regardless of the costs. However, blind functional assessment does not reveal insights to container content.

Modular Assessment
An enhancement to functional assessment would be having the ability to associate different choices of software and protocol to the differences in outcomes that we see. For example, knowing the exact location of executables and data to produce each output of "Hello World," we can perform further comparisons on the contents, or make the calls language agnostic by doing a trace of system calls [15]. For this kind of assessment to be possible, container organization and accessibility is paramount.

Collaborative Assessment
As another example, imagine that a single container provides ten different implementations of a sorting algorithm in Python. Running any one of the algorithms, each a SCI-F app, would occur on the same input data, and the same host inside the container. Given a pre-defined metric to assess each result, we might programatically parse over the Python scripts and compare imports and functions used across sorting algorithms. This is the idea of a Collaborative (or Competitive) container, which has two key components:

i. a function and output of interest
ii. a metric of goodness to assess the output

A scientist wanting to answer a specific scientific question can then approach answering his question by asking other scientists to write SCI-F apps. Each contribution is then assessed by the pre-defined metric of goodness, or assessed as the analysis code changes over time [16]. All of this might occur collaboratively with version control (e.g. Github [17]) linked to a Continuous Integration [5] testing environment to run the contribution and make the assessment. SCI-F would make this possible.

Structure of SCI-F

We now move into describing the standard itself. The Standard Container Integration Format is a set of rules about how a container software installs, organizes, and exposes software modules. We will start with a review of some basic background about Linux Filesystems.

Traditional File Organization

File organization is likely to vary a bit based on the host OS, but arguably most Linux flavor operating systems can said to be similar to the Filesystem Hierarchy Standard (FHS) [7]. For this discussion, we will disregard the inclusion of package managers, symbolic links, and custom structures, and focus on the core of FHS. We will discuss these locations in the context of how they do (or should) relate to a scientific container. It was an assessment of this current internal standard that led to the original development of SCI-F.

Do Not Touch
Arguably, the following folders should not be touched by scientific software:

- **/boot**: boot loader, kernel files
- **/bin**: system-wide command binaries (essential for OS)
- **/etc**: host-wide configuration files
- **/lib**: system level libraries
- **/root**: root's home [6]
- **/sbin**: system specific binaries
- **/sys**: system, devices, kernel features

While these locations likely have libraries and functions needed by the host to support software, it should not be the case that a scientist installs his or her software under any of these locations. It would not be easy or intuitive to find it hidden with what is already provided by the host.

Variable and Working Locations
The following locations are considered working directories in that they hold variables defined at runtime, or intermediate files that are expected to be purged at some point:

- **/run**: run time variables for running of programs.
- **/tmp**: temporary location for users and programs
- **/home**: can be considered the user's working space [7]

Connections
Connections for containers are devices and mount points. A container will arguably always need to be able to support mount points that might be necessary from its host, so it would be important for a scientific container to not put valuables in these locations.

- **/dev**: essential devices
- **/mnt**: temporary mounts.
- **/srv**: is for "site specific data" served by the system. This might be a logical mount for cluster resources.
- **/proc**: connections between processes and resources and hardware information

SCI-F File Organization

The Standard Container Integration Format defines two root bases that can be known and consistently mounted across research clusters. These locations were chosen to be independent of any locations on traditional linux filesystems for the sole purpose of avoiding conflicts.

Apps
The base of /scif/apps is where software modules reside. In the following examples, We describe interaction with apps and the file system structure via the implementation in the Singularity [9] software. Singularity builds containers from a build specification file called a Singularity Recipe. It is a text file with different sections, each filled with commands for the build process to execute. In the example below, we create a build recipe to start with a base Docker image, ubuntu, and install a hypothetical app "foo" into it:

```
Bootstrap: docker
From: ubuntu:latest

%post
    apt-get update

%appinstall foo
    git clone ...
    cd foo-master
    ./configure --prefix=../bin
    make
    make install
```

5 https://www.cirleci.com or https://www.travis-ci.org
6 Unless you are using Docker, putting things here leads to trouble.
7 Singularity mounts the user's home by default, and would override anything in the container's /home. For these reasons, it is not advisable to assume stability in putting software in these locations.

```
%applabels foo
MAINTAINER vsochat@stanford.edu
FOO_VERSION 9.19

%appenv foo
FOO=BAR
export FOO

%appfiles foo
README.md README.md
run_tests.sh bin

%apptest foo
/bin/bash run_tests.sh

%apphelp foo
Foo: will produce you bar.
Usage: foo [action] [options] ...
 --name/-n name your bar

%apprun foo
    exec foo
```

In the example above, we defined an application (app) called "foo" and gave the container sections for it, including a recipe for installation (%appinstall), a simple text print out to show to some user that wants help (%apphelp), tests to validate that it runs correctly (%apptest), and an entry point script to call when the user executes the container asking for foo (%apprun). We add files in the %appfiles section, and labels to describe foo in %applabels. For installation and files, paths are relative to the install folder.

Apps Sections

Based on the section name (e.g. "%apprun foo"), the specifics of each section are defined as follows:

%appinstall. corresponds to executing commands in the context of the app's install folder to install the application. These commands would previously belong in %post, but are now attributable to the application.

%apphelp. is written as a file called "runscript.help" in the application's metadata folder, where the Singularity software knows where to find it. If no help section is provided, the software simply will alert the user and show the files provided for inspection.

%apprun. is written as a file called "runscript.exec" in the application's metadata folder, and again looked for when the user asks to run the software.

%applabels. will write a labels.json in the application's metadata folder, allowing for application specific labels.

%appfiles. copies files from the host to the application install folder. For example, specifying the file README.md for foo will produce the file /scif/apps/foo/README.md in the container.

%appenv. will write an environment file in the application's base folder, allowing for definition of application specific environment variables.

%apptest. will run tests specific to the application, with present working directory assumed to be the software module's folder

Apps Generation

The Singularity software would do the following based on this set of instructions:

- Finding any app section alongside a name (e.g., %appinstall foo) is indication of an application command.
- The name string (e.g. foo) is parsed as the name of the application, and a folder is created, in lowercase, under /scif/apps if it doesn't exist.
- The installation procedure (%appinstall) is performed to install dependencies in the container relative to the app's install folder.
- A metadata folder, scif, is generated inside the application folder.
- The environment, help, runscript, and labels provided for "foo" in the build recipe are saved to the metadata folder.
- A "bin" folder is automatically generated for foo, and will be automatically added to $PATH when foo is being used. A "lib" folder is also generated, and is added to $LD_LIBRARY_PATH when foo is used.
- If the (%apptest) section is defined, tests are run on the newly installed app to insure it works as expected.

Data

The base of /scif/data is structured akin to apps - each installed application has its own folder, and additionally a subfolder is created for inputs and outputs:

```
/scif/data
   /foo
      /input
      /output
```

SCI-F does not enforce or state how the container creator should use the data folders, but rather encourages the creator to use the organization so that a user can intuitively know that any input for app foo might go into /scif/data/foo/input, general data for foo might be in /scif/data/foo, and global data for the entire container might be in /scif/data. The latter would mean that a scientific container could mount a host folder to /scif/data, and then generated results are generated at /scif/data during the running of each SCI-F app, which can find their data bases by referencing an environment variables [8]. As container functions and integrations are further developed, we expect this simple connection into a container for inputs and outputs specific to an application to be very useful. As for the organization and format of the data for any specific application, this is up to the application. Data can either be included with the container, mounted at runtime from the host, or provided statically and used internally without any mount.

Data Modularity

Akin to software modules, overlap in data modules is not allowed by way of the unique app names afforded by folders under a common directory. For example, let's say we have an application called "foo".

- users and developers would know that foo's data would be mounted or provided at /scif/data/foo.
- importing of datasets within an app's data folder that follow some other specific format [18] would be allowed, e.g., /scif/data/foo/bar1 and /sci/data/foo/bar2.
- An application's data would be traceable to the application by way of it's identifier. Thus, if I find /scif/data/foo I would expect to find related software under /scif/apps/foo.

8 for example, when app "foo" is active, the environment variable SINGULARITY_APPDATA references /scif/data/foo

Environment Variables

Discovery of data and app folders is helped by way of environment variables. When the container is run in context of an app:

```
$ singularity run --app foo container.simg
```

a set of environment variables about locations for the app's data and executables are exposed. Variables are also exposed for these locations for other apps, and both these sets of variables make it easy for the creator and user to reference locations without knowing the actual paths. Thus, a container with SCI-F apps provides the following (automatically generated) runtime environment variables that can be used in build recipes. Given the implementation in Singularity containers, these variables are prefixed appropriately:

- **SINGULARITY_APPS**: An environment variable to point to the global apps base (/scif/apps)
- **SINGULARITY_DATA**: the global data base (/scif/data)
- **SINGULARITY_APPDATA**: references the app that is being run (e.g., foo), pointing to an app's data base (/scif/data/foo)
- **SINGULARITY_APPINPUT**: inputs (/scif/data/foo/input)
- **SINGULARITY_APPOUTPUT**: outputs (/scif/data/foo/output)

The next set of variables are defined for every app, regardless of the currently active app. This makes it possible to know the path to another app's data (e.g. bar) while running foo.

- **APPROOT_<bar>**: defined as (**/scif/apps/bar**)
- **APPDATA_<bar>**: defined as (**/scif/data/bar**)

While SCi-F is not a workflow manager, it follows naturally that the creator of a SCI-F app might use these internal variables to have modules internally talk to one another. The user and creator do not need to know the structural specifics of the standard, but only how to reference them.

Interaction

A important feature of container software applications is allowing for programmatic accessibility to a specific application within a container. For each of the Singularity software's main commands (run, exec, shell, inspect and test) the same commands can be easily run for an application.

Listing Applications
If I wanted to see all applications provided by a container, I could use singularity apps:

```
$ singularity apps container.simg
bar
foo
```

Application Run
To run a specific application (defined in the %apprun section of the Singularity recipe), I can use run with the "app" flag:

```
$ singularity run --app foo container.simg
RUNNING FOO
```

In the case that an application doesn't have a runscript, the default action is taken, shelling into the container:

```
$ Singularity run --app bar container.simg
No Singularity runscript found, executing /bin/sh
Singularity>
```

Application Execution and Testing
For the commands shell and exec, in addition to the base container environment being sourced, if the user specifies a specific application, any variables specified for the application's custom environment are also sourced. A container with an application that had tests defined in its %apptest section can also be tested.

```
$ singularity test --app bar container.simg
Application Testing for bar...
```

Container Inspection
In the case that a user wants to inspect a particular application for a runscript, test, or labels, that is possible on the level of the application:

```
singularity inspect --app foo container.simg
{
    "SINGULARITY_APP_SIZE": "1MB",
    "SINGULARITY_APP_NAME": "foo",
    "MAINTAINER": "vsochat@stanford.edu",
    "FOO_VERSION": "9.19"
}
```

The above shows the default output, the labels specific to the application foo. The user can also ask for a snippet of help text for a single app:

```
$ singularity help --app foo container.simg
Foo: will produce you bar.
Usage: foo [action] [options] ...
 --name/-n name your bar
```

Metadata about dependencies and steps to create the software would be represented in the %appinstall, which is by default saved with each container. Metadata about different environment variables would go into %appenv, and labels that should be accessible statically go into %applabels. Help for the user is provided under %apphelp.

Example Use Cases

SCI-F is powerful in that it supports multiple general use cases for scientific and systems evaluation and high level introspection. These use cases broadly fall in the areas of providing modular software, systems and metric evaluation, and guided collaboration to answer a scientific question.

Modular Software Evaluation

A common question pertains to evaluation of different solutions toward a common goal. An individual might ask "How does implementation A compare to implementation B as evaluated by one or more metrics?" For a systems admin, the metric might pertain to running times, resource usage, or efficiency. For a researcher, he or she might be interested in looking at variability (or consistency) of outputs. Importantly, it should be possible to give a container serving such a purpose to a third party that does not know locations of executables, or environment variables to load, and the container runs equivalently. SCI-F allows for this by way of providing modular software applications, each corresponding to custom environments, li-

Figure 1. Assessing "read calls" across a range of different programming language implementations of "Hello World" shows a surprising range of differences, and reflects common knowledge that more extensive programs (e.g., Octave) add complexity to the seemingly simple command.

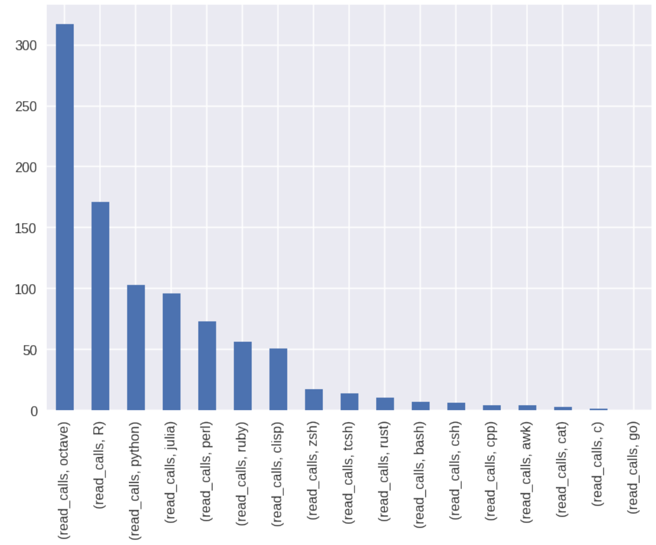

braries, and potentially files.

Method

To demonstrate this use case, we developed a container that implements the most basic function for a program, a print to the console, for each of 19 different languages (R, awk, bash, c, cat, chapel, clisp, cpp, csh, go, julia, octave, perl, python, ruby, rust, tcsh, zsh). The container is designed as a means to collect a series of metrics relative to timing and system resources for each language. The metrics pertain to system resources provided by the time [19] and strace [15] utilities. The evaluation can be run across modules (languages), without knowing the applications installed with a simple for loop.

```
for app in $(singularity apps hello-world.simg)
    do
    singularity run --app $app hello-world.simg
done
```

Results

To demonstrate the value of using SCI-F containers, we collected run time metrics for 19 SCI-F apps, each of which printed "Hello World" using a different programming language. We were able to run the analysis in entirety without knowing the specific commands for each language. The resulting table of features pertaining to times and features demonstrates a wide span of differences between the seemingly identical calls. For example, Figure 1 shows the differences in “read calls,” or the number of read commands to the file system issued when the simple “Hello World” command was run.

Closer inspection reveals facts about the programs that are common knowledge, such as shell programs having faster start up times than more substantial programs (e.g., octave, R, or Python). In fact, the basic differences between start times, reads and writes, and memory usage across this simple execution is surprising, and gives strong support for why scientific results can vary depending on the underlying architecture. It gives even stronger rationale for being able to assess the metadata about the software to reveal cause for the observed differences. Full results and additional analyses are available in this notebook, and a more detailed writeup is provided. The full analysis code and results are also provided.

Modular Metrics Evaluation

For this next use case, a scientist is interested in running a series of metrics over an analysis of interest (the container’s main function, executed by its primary runscript). He has been given a container with a runscript, and several installed supporting metrics (SCI-F apps also in the container), and knows nothing beyond that.

Each installed SCI-F app can be thought of as a particular context to evoke the container’s main runscript, and the apps themselves are relatively agnostic to the runscript itself. Importantly, using the image for its intended purpose is not impacted by the presence of these supporting tools. The command to run the image is unchanged. When the scientist runs the image, he sees it perform it’s primary function, a print of “Hello World!” to the console. He can discover the metrics provided with the "singularity apps" command, and then run a named metric easily by simply specifying it,

```
$ singularity run --app time metrics.img
```

This particular container has several metrics to assess usage and timing of different resources (time), a complete trace of the call (strace), a static linter (linter), and a function to run the container’s runscript in parallel (parallel). Each of these SCI-F apps serves as an example use case that is discussed in the following sections.

Metric Example 1: Evaluate software across different metrics

A system admin or researcher concerned about evaluation of different software could add relevant metrics apps to the software containers, and then easily evaluate each one with the equivalent command to the container. Importantly, since each evaluation metric is a modular app, the container still serves its intended purposes. As an example, here is a simple app to return a table of system traces for the runscript:

```
%apprun strace
    unset SINGULARITY_APPNAME
    exec strace -c -t /.singularity.d/actions/run
```

In the above example, since the main run command for the container looks for the SINGULARITY_APPNAME (and we have already called it), we need to unset it first to target the container’s primary runscript. We then run strace and return a table that assesses the runscript. The interaction with the user looks like the following:

```
singularity run --app strace metrics.img
Hello-World!

% time     seconds  usecs/call     calls    errors syscall
------ ----------- ----------- --------- ---------
       ----------------
  0.00    0.000000           0        15           read
  0.00    0.000000           0         1           write
  0.00    0.000000           0        35        24 open
  0.00    0.000000           0        17           close
  0.00    0.000000           0        25        12 stat
  0.00    0.000000           0         4           fstat
  0.00    0.000000           0        14           mmap
  0.00    0.000000           0         8           mprotect
  0.00    0.000000           0         2           munmap
  0.00    0.000000           0         6           brk
  0.00    0.000000           0        14           rt_sigaction
  0.00    0.000000           0         6         6 access
  0.00    0.000000           0         2           getpid
```

```
 0.00    0.000000           0         2           execve
 0.00    0.000000           0        14           fcntl
 0.00    0.000000           0         2           getdents
 0.00    0.000000           0         3           geteuid
 0.00    0.000000           0         2           getppid
 0.00    0.000000           0         2           arch_prctl
 0.00    0.000000           0         1           openat
 0.00    0.000000           0         1           faccessat
------ ----------- ----------- --------- ---------
      ----------------
100.00    0.000000                   176        42 total
```

Regardless of what the runscript does, this SCI-F app will provide a consistent way to produce this metric. Any user that added the small module to his or her container would immediately have this assessment for the software provided by his or her container. The recipe for this strace app is provided at the containers-ftw apps portal (https://www.containers-ftw.github.io/apps), discussed in more detail later in this document.

Metric Example 2: Code Quality and Linting

A SCI-F app can meet the needs to serve as a linter over a set of files. The example is provided here with a SCI-F app "linter," which runs a linter over a script.

```
$ singularity run --app linter metrics.simg

In /scif/apps/linter/lintme.sh line 2:
for f in do;
^-- SC2034: f appears unused. Verify it or export it.
         ^-- SC1063: You need a line feed or semicolon before
              the 'do'.
           ^-- SC1059: No semicolons directly after 'do'.


In /scif/apps/linter/lintme.sh line 3:
grep -qi hq.*mp3 && echo -e 'Foo bar'; done
        ^-- SC2062: Quote the grep pattern so the shell won't
             interpret it.
                       ^-- SC2039: #!/bin/sh was specified, but
                            echo flags are not standard.
```

This example used a file provided in the container, but a linter app could also accept a command line argument to a file or folder. Note that a testing function that is run as an app is not equivalent to a test for the container during build. We advise the researcher to still use the %test section to evaluate the outcome of the build process, and to use SCI-F apps for tests that are generalizable as tools.

Metric Example 3: Runtime Evaluation

In that a metric can call a runscript, it could be easy to evaluate running a primary analysis under various conditions. As a simple proof of concept, here we are creating an app to execute a script in parallel.

```
%apprun parallel
    unset SINGULARITY_APPNAME
    COMMAND="/.singularity.d/actions/run; "
    (printf "%0.s$COMMAND" {1..4}) | parallel

$ singularity run --app parallel metrics.simg
Hello World!
Hello World!
Hello World!
```

And you might imagine a similar loop to run an analysis, and modify a runtime or system variable for each loop, and save or print the output to the console.

This metrics implementation is available. [9]. Complete description and documentation is available. [10]

Contextual Running

It's often common that a user will want to run a container in different environments, as is the case with launching a container to run using different job managers. For example, a scientific analysis run locally would come down to executing the script, but run on a cluster would come down to submission of a job to a SLURM [20] or SGE [21] queue. In this case, a scientist could distribute the image with easy entry points to each of these use cases, and the container is easy to run in multiple environments.

```
$ singularity run --app slurm analysis.simg
$ singularity run --app sge analysis.simg
```

During the build process, if the resources are available, the researcher can measure metrics like memory needed and time, and then write them into the batch job. Runtime variables like queue and notification email could be provided via variables to the runscript. This particular example for slurm and sge have been implemented. [11] [12]

A cluster that provides containers for its users could provide submission scripts for optimal resource usage for each container.

Scientific Workflows

Distributing scientific workflows is arguably the most common use case for scientific containers. A scientist is likely to use SCI-F apps for two purposes:

- to provide development containers that expose software to test and develop pipelines
- to provide a production container alongside a publication to serve a final pipeline

SCI-F can meet both of these goals, and for this example, we have implemented the equivalent pipeline using Singularity and SCI-F for the CarrierSeq workflow [22], as well as adding SCI-F to a previously done variant calling analysis that used Singularity and Docker [13] Each of the two example containers provides modular access to the different software inside. By way of using the Standard Container Integration Format, we have a lot of freedom in deciding on what level of functions we want to expose to the user. A developer will want easy access to the core tools (e.g., bwa, seqtk) while a user likely wants one level up, on the level of a collection of steps associated with some task (e.g., mapping).

Carrierseq Scientific Pipeline

For this example, we focus on the build recipe that generates a scientific container to perform a download of input data (optional, as the user may already have a dataset of interest), a mapping, a statistical procedure (poisson), and a sorting procedure. We assume that an interested party has found the con-

9 https://github.com/containers-ftw/metrics-ftw
10 http://containers-ftw.org/apps/examples/metrics/metrics-ftw.
11 http://containers-ftw.org/apps/scif/hpc/sge/hpc-sge-submit/
12 http://containers-ftw.org/apps/scif/hpc/sge/hpc-slurm-submit/
13 https://github.com/vsoch/singularity-scientific-example.

tainer "cseq.simg", has Singularity installed, and is curious about how to use it. The individual could first ask for help directly from the container.

```
$ singularity help cseq.simg

    CarrierSeq is a sequence analysis workflow for low-input
          nanopore
    sequencing which employs a genomic carrier.

    Github Contributors: Angel Mojarro (@amojarro),
                         Srinivasa Aditya Bhattaru (@sbhattaru),
                         Christopher E. Carr (@CarrCE),
                         Vanessa Sochat (@vsoch).

    fastq-filter from:
          https://github.com/nanoporetech/fastq-filter
    see:
           singularity run --app readme carrierseq.img | less
                 for more detail
```

If we follow the instruction, we find the container has an APP that serves only to make the README.md easily accessible:

```
$ singularity run --app readme cseq.simg | less

#### CarrierSeq
#### About

bioRxiv doi: https://doi.org/10.1101/175281

CarrierSeq is a sequence analysis workflow for low-input
      nanopore
            sequencing which employs a genomic carrier.

           Github Contributors: Angel Mojarro (@amojarro),
                                Srinivasa Aditya Bhattaru
                                      (@sbhattaru),
                                Christopher E. Carr (@CarrCE),
                                and Vanessa Sochat (@vsoch).

fastq-filter from: https://github.com/nanoporetech/fastq-filter

[MORE]
```

Metadata in the way of labels, environment, help, and the runscript and build recipes themselves are available for the whole container in either a json [23] or human readable format via the –inspect command:

```
$ singularity inspect cseq.simg
{
    "org.label-schema.usage.singularity.deffile.bootstrap":
          "docker",
    "org.label-schema.usage.singularity.deffile": "Singularity",
    "org.label-schema.usage": "/.singularity.d/runscript.help",
    "org.label-schema.schema-version": "1.0",
    "org.label-schema.usage.singularity.deffile.from":
          "ubuntu:14.04",
    "org.label-schema.build-date": "2017-09-20T18:16:50-07:00",
    "BIORXIV_DOI": "https://doi.org/10.1101/175281",
    "org.label-schema.usage.singularity.runscript.help":
          "/.singularity.d/runscript.help",
    "org.label-schema.usage.singularity.version":
          "2.3.9-development.gaaab272",
    "org.label-schema.build-size": "1419MB"
}
```

and also available on the level of individuals apps:

```
$ singularity inspect --app mapping cseq.simg
{
    "FQTRIM_VERSION": "v0.9.5",
    "SEQTK_VERSION": "v1.2",
    "BWA_VERSION": "v0.7.15",
    "SINGULARITY_APP_NAME": "mapping",
    "SINGULARITY_APP_SIZE": "9MB"
}
```

All apps are exposed to the user:

```
$ singularity apps cseq.simg
mapping
poisson
readme
sorting
download
```

And then the user can ask for help for any of the pipeline steps:

```
$ singularity help --app mapping cseq.simg
$ singularity help --app poisson cseq.simg
$ singularity help --app sorting cseq.simg
```

The entire set of steps for running the pipeline provided by the container comes down to calling the different apps. As an overall strategy, since the data is rather large, we are going to map a folder to the container's data base where the analysis is to run. This directory, just like the modular applications, has a known and predictable location. So our steps are going to look like this:

i. Download data to a host folder
ii. For subsequent commands, map /scif/data to the host
iii. Perform mapping step of pipeline
iv. Perform poisson regression on filtered reads
v. Sort the results

And the calls to the container to support this would be:

```
$ singularity run --app mapping --bind data:/scif/data cseq.simg
$ singularity run --app poisson --bind data:/scif/data cseq.simg
$ singularity run --app sorting --bind data:/scif/data cseq.simg
```

This would be enough to run the pipeline. What do the modules afford us? We can easily isolate metadata and contents related to each step, or shell into the context to test:

```
$ singularity shell --app mapping cseq.simg
```

We might also decide that we don't like the "mapping" step, and swap it out for one provided by a different container.

```
$ singularity run --app mapping --bind data:/scif/data map.simg
$ singularity run --app poisson --bind data:/scif/data cseq.simg
$ singularity run --app sorting --bind data:/scif/data cseq.simg
```

A researcher that is incredibly interested in variations of one step (e.g., sorting) could provide an entire container just to serve those variations, to then be used with carrierseq:

```
S singularity run --app quick --bind data:/scif/data sort.simg
$ singularity run --app merge --bind data:/scif/data sort.simg
```

Importantly, metadata and container contents relevant to a specific step (e.g., "mapping") are represented in the build recipe (the record of instructions that originally built the container and defined the apps) and the content of the filesystem itself (e.g., /scif/apps/mapping). The examples above show that SCI-F provides a standard set of commands that could in-

tegrate easily into a workflow manager, but also expose intuitive entry points for users that may not have expertise to use such a manager. The creator of the container, the scientist, can carefully craft commands to be specific to his work, and the user is not expected to know the trivial details to use it. In fact, exposure to the details may even be a detriment if it confuses the user. This is a very different use case from a scientific developer's, discussed next.

Carrierseq Development Container

The developer has a different use case - to have easy command line access to the lowest level of executables installed in the container. Given a global install of all software, without SCI-F I would need to look at $PATH to see what has been added to the path, and then list executables in path locations to find new software installed to system locations like /usr/bin. I could only assume that the creator of the container thought ahead to add these important executables to the path at all. Unfortunately, there is no way to easily and programmatically "sniff" a container to understand what changes were made, and what tools are available for development. A container created by developer Sam is likely not going to be understood by developer Stan. We would do well to create a development container with SCI-F, and for this discussion, have created such a build recipe. [14]

For the CarrierSeq development container, instead of serving software on the level of the pipeline, we reveal the core software and tools that can be combined in specific ways to produce a pipeline step like "mapping."

```
$ singularity apps dev.simg
bwa
fqtrim
python
seqtk
sra-toolkit
```

Each of the above apps can be used with commands "run", "exec," "inspect," "shell," or "test" to run the container in context of a particular app. This means sourcing app-specific environment variables, and adding executables associated with the app to the path. For example, I can use a simple app "python" to open the python interpreter in the container, or shell into the container to test bwa:

```
##### Open interactive python
$ singularity run --app python dev.simg
>>

##### Load container with bwa on path
$ singularity shell --app bwa dev.simg
$ which bwa
$ /scif/apps/bwa/bin/bwa
```

These two CarrierSeq images that serve equivalent software, but enable very different use cases, are good example of the flexibility of SCI-F. The container creator can choose the level of detail to expose to a user that doesn't know how it was created. A lab that is using core tools for working with sequence data might have preference for the development container, while a finalized pipeline distributed with a publication would have preference for the first.

14 https://github.com/vsoch/carrierseq

Singularity Scientific Example

Finally, we adopted an original analysis [15] to compare Singularity vs. Docker on different cloud and local environments to give rationale for taking a SCI-F apps approach over a traditional Singularity image. We compare same pipeline implemented with SCI-F, and without SCI-F as an example of how containers can provide the same software to perform the same function, but notably, have different organization that impacts discoverability. A detailed writeup of the rationale and use case is provided for the reader, along with the code base for the container [16]. In summary, Singularity without SCI-F relies on external scripts and the container is a black box. Singularity with SCI-F has no external dependencies beyond data, and communicates its usage clearly.

Research Evaluation

Containers aren't only useful for running scientific pipelines, they are sources of information to discover good practices and features of scientific software. Having modular software apps allows for separation of files and executables for research from those that belong to the base system, enabling this kind of research. From a machine learning standpoint, the apps and corresponding metadata provide labels for a supervised algorithm to compare between apps and containers. In addition to the filesystem under /scif, the build recipe might also be parsed to see what software (possibly outside of the /scif root location) was intended for each app. Equally important, when apps are installed, having container software installed at a global at %post suggests that the software is globally important.

Working Environments

SCI-F has a very interesting use case when it comes to working environments. Each app defined in a container can be thought of as running the container under a different context. Given that each app is associated with its own environment, labels, and executables on the $PATH, a container can serve custom working environments. Imagine that the execution of some command is not the goal of the container, but rather providing a set of environment around a software core. There is no minimum required set of sections to define an app, so a container that is intended as a "working container" might simply be a set of %appenv sections to define different named environments. Without any other section, the user is then able to interact with the custom, named environments.

```
$ singularity shell --app tensorflow-gpu container.simg
```

Auditing and Logging

Although we do not delve into this use case, it should be noted that SCI-F apps can provide logging and auditing for containers. A systems administration that builds and provides containers for his or her users might want to enforce running with a standard for logging and auditing [24]. Instead of asking the researcher to write this into his or her custom runscript, the snippet to perform the logging could be added as a SCI-F app dynamically at build time, and then the container run with this context.

15 https://github.com/vsoch/singularity-scientific-example
16 https://github.com/containers-ftw/scientific-example-ftw

Community

To encourage sharing and distribution of useful apps, we have developed an online interface for easily exploring and sharing SCI-F apps, and generating recipes using the apps, available at https://containers-ftw.github.io/apps.

Community Infrastructure

The interface is served from a Github repository that renders static template files into a complete website that includes search across all content, exploration by tag (e.g., language or operating system), and instruction by way of reading examples and tutorials. Programmatic access to all apps is provided with a RESTful API, as is an feed for interested users to be notified when new content is added. The interface also includes a recipe generator that allows a user to browse the site, save apps of interest in the browser's local storage, and then combine them in a Singularity build file that can be downloaded in a compressed archive that includes instructions and any associated, required files for the app.

The Singularity container registry Singularity Hub [17] provides a build service for the Singularity community, and is designed to automatically extract complete metadata about apps that it discovers in containers. The metadata including app names, environments, and labels is indexes and search-able on the Singularity Hub site. These tools, along with the ease of using SCI-F, will greatly improve container transparency and recipe sharing.

Contributing

Importantly, as the infrastructure is served from a Github repository, contributing does not require any expertise with web development or related technologies. The user can simply use Github to fork the repo, add a text file to a folder (_apps), and submit a pull request (PR) to evaluate the contribution. The text file itself has a header section that contains bullet pointed lists of metadata like name, tags, and files, and the remainder of the file is the Singularity sections for the app (e.g., %apprun hello-world). When the PR is approved, the contribution is automatically rendered into all areas of the community site. If an app includes associated files like scripts or configuration, this data is also easily added into a folder named equivalently to the file, alongside it (e.g., _apps/hello-world/hello-world-bash.md would have associated files in _apps/hello-world/hello-world-bash). By way of using version control, all changes and contributions are tracked and credit allocated.

Testing

Github also allows for complete testing of all contributions, and the repository is set up with a continuous integration (testing) service called CircleCI [18] that checks the following:

- The file name for the app corresponds with the app's name declared in the file
- The folder path under _apps also corresponds to the app's file name. For example, an app located at _apps/hello-world/bash/ must start with hello-world-bash. Matching app names to the folder structure ensures uniqueness of the names within the repository.
- The user has not provided any empty keys or values.
- Each declared file is included in the repository
- The app minimally has a tag for one operating system [19].
- The header date is in valid format to be rendered correctly
- Fields allowed in the header do not go beyond "author," "title," "date," "files," and "tags.
- Required fields ("author," "title," "date," and "tags") are present

Any contribution that does not meet these requirements will get feedback during the PR, and the contributor can adjust the file to address any issues. As soon as the content is merged into the master branch, it is immediately live on the site. The following are examples for the utility of this resource:

- A user can find useful examples and apps for his or her Singularity containers.
- A contributor can easily improve SCI-F via a pull request, or an issue that suggests change.
- A user can contribute to the Singularity software that has the SCI-F implementation.
- The user can contribute an app for others to use.
- The user can, most simply, ask a question or get help

Future Work

SCI-F is exciting because it makes basic container development and usage easier. The user can immediately inspect and see the software a container provides, and how to use it. The user can install additional software, use an app in a different container, or view metadata and help documentation. The developer is provided guidance for how and where to install and configure software, but complete freedom with regard to the software itself, and the level of modularity to expose. The minimum requirements for any package are a unique name within the container, and then any one of the needed sections. In addition to these basic examples, we provide other future use cases that would be possible with the Standard Container Integration Format.

Mapping of container landscape

Given separation of the software from the host, we can more easily derive features that compare software modules. These features can be used with standard unsupervised clustering to better understand how groups of software are used together. We can further apply different labels like domains and understand what modules are shared (or not shared) between scientific domains. We can find opportunity by discovering gaps (for example, that a software module isn't used for a particular domain) and then question why this is the case.

Artificial Intelligence (AI) Generated Containers

Given some functional goal, and given a set of containers with measurable features to achieving it, we can (either by brute force or more elegantly) procedurally generate and test containers toward some metric. The landscape of containers can easily be pruned in that the best containers for specific use cases can be easily determined automatically. This is, abstractly, a new kind of operating system that essentially designs itself [25].

17 https://www.singularity-hub.org
18 https://www.circleci.com
19 to help determine compatibility

Discussion

In summary, SCI-F is useful because it allows for:

- flexible, internal **modularity** where the definition of modularity is entirely based on the needs of the creator and user, and the resulting container reflects that.
- **reproducible practices** by way of providing portable environments with modular internal contents that are easily discovered.
- **integration** with external tools.
- **predictable internal structure** that distinguishes scientific content from the operating system base.
- **community resources** including APIs, version control and testing, and open forums for tracking issue and discussion related to SCI-F and SCI-F apps.

This discussion would not be complete without a mention of limitations, and suggested best practices.

Limitations

It is important to distinguish the entire container as a reproducible product, and different software modules inside of it that are served by the container. While the container itself is portable, and designed to contain all dependencies to support reproducibility, a SCi-F module in and of itself is not guaranteed to be. For example, a user might define a module only with an %apprun section, implying that the folder only contains a runscript to execute. The user may have chosen to install dependencies for this script globally in the container (in the %post section) because perhaps they are shared across multiple modules. Under these conditions, if another user expected to add the module to a different build recipe, the dependencies from %post would be needed too. The host operating system also needs to be taken into consideration. A module with dependencies installed from the package manager "yum" would not move seamlessly into a Debian base. However, appropriate checks and balances can be implemented to help with movement of apps between containers.

Best Practices

App Installation

To avoid missing dependencies, users are encouraged to include all dependency installs within the %appinstall section to make their apps maximally portable outside of their initial container. It's also good practice to use the %apptest section to ensure that an app that might have been added to a new container is functioning as it should. Finally, metadata should be provided with apps about points of contact, usage and documentation, and supported operating system bases. To encourage this practice, we have added a test and requirements of specifying one or more operating systems for any module contributed at https://containers-ftw.github.io/apps.

Global vs App Install

In the case of software that can be installed globally using a package manager (e.g., Python), it is up to the creator to decide if a global versus. app install is more appropriate. In practice, we have found that global installs tend to be larger, well maintained libraries (e.g., libraries installed with apt-get or package managers like pip), and having them installed in %post, to be shared among apps, is most appropriate. In the case of wanting multiple versions of the same software, an App install is most appropriate to keep the environments isolated. This decision is up to the generator of the container.

We do not enforce using SCI-F for Singularity images or any other container. Its creation and discussion is implemented and provided to only help scientists more easily create reproducible, transparent containers.

Conclusion

We have presented the Standard Container Integration Format and have shown examples of its functionality for container interaction, development, and scientific pipelines. The Standard Container Integration Format is advantageous in that the container creator can embed his or her work with implied metadata about software and container contents. SCI-F also makes it easier to package different runscripts with the container, and expose them easily to the user. However, this does not mean that the standard approach of using a container as a general toolbox and distributing it with a series of external callers is bad or wrong. The choice to use (or not use) SCI-F apps is largely dependent on the goals of the creator, and the intended users. We hope that SCI-F is useful for the larger community, and encourage contribution and asking questions.

Appendix

Resources

The following is a list of (possibly) related standards, formats and initiatives.

- File Hierarchy Standard
- Open Containers Initiative
- Common Workflow Language
- Fair Principles
- Open Standards
- https://reproducible-builds.org/
- DASPOS: https://daspos.crc.nd.edu
- TANGO: http://tango-project.eu/

Code Availability

The Standard Container Integration Format is implemented into the the Singularity software, which is open source and freely available.

- Project name: Singularity
- Project home page: e.g. `https://singularityware.github.io`
- Operating system(s): e.g. Linux
- Programming language: e.g. C,python,bash
- License: e.g. BSD 3 Clause

Availability of supporting data and materials

The software and code supporting the use cases in this article is(are) available in the several code repositories.

- SCI-F Implementation https://singularityware.github.io/singularity
- SCI-F Documentation https://containers-ftw.github.io/SCI-F/
- SCI-F Apps and Resources https://containers-ftw.github.io/apps/

Declarations

Ethical Approval

Not applicable

Consent for publication

Not applicable

Competing Interests

The author(s) declare that they have no competing interests.

Funding

V. Sochat is supported by the Stanford Research Computing Center and the Stanford School of Medicine.

Author's Contributions

V. Sochat conceptualized, implemented, developed, and tested the Standard Container Integration Format, along with the associated web applications, examples, and tutorials.

Acknowledgements

V. Sochat would like to thank the community for substantial feedback on the standard and the manuscript draft. Specifically, thanks to Pim Schravendijk, Ruth Marinshaw, Satra Ghosh, Remy Dernat, Gregory Kurtzer, and Paolo D. Tommasso for feedback on the draft and standard. Special thanks to the larger Singularity Community for making the open source project fun, needed, and inspiring.

References

1. Glatard T, Lewis LB, Ferreira da Silva R, Adalat R, Beck N, Lepage C, et al. Reproducibility of neuroimaging analyses across operating systems. Front Neuroinform 2015 Apr;9.
2. Merkel D. Docker: Lightweight Linux Containers for Consistent Development and Deployment. Linux J 2014 Mar;2014(239).
3. Docker-based solutions to reproducibility in science - Seven Bridges; 2015. Accessed: 2016-12-17. https://blog.sbgenomics.com/docker-based-solutions-to-reproducibility-in-science/.
4. Hosny A, Vera-Licona P, Laubenbacher R, Favre T. AlgoRun: a Docker-based packaging system for platform-agnostic implemented algorithms. Bioinformatics 2016 Aug;32(15):2396–2398.
5. Moreews F, Sallou O, Ménager H, Le Bras Y, Monjeaud C, Blanchet C, et al. BioShaDock: a community driven bioinformatics shared Docker-based tools registry. F1000Res 2015 Dec;4:1443.
6. Boettiger C. An introduction to Docker for reproducible research, with examples from the R environment 2014 Oct;.
7. Linux Filesystem Hierarchy;.
8. Wikipedia contributors, Comparison of file systems; 2016. Accessed: 2016-11-23. https://en.wikipedia.org/w/index.php?title=Comparison_of_file_systems&oldid=751048657.
9. Kurtzer GM, Sochat V, Bauer MW. Singularity: Scientific Containers for Mobility of Compute;.
10. Overview of Docker Compose;. Accessed: 2016-1-9. https://docs.docker.com/compose/.
11. Di Tommaso P, Palumbo E, Chatzou M, Prieto P, Heuer ML, Notredame C. The impact of Docker containers on the performance of genomic pipelines. PeerJ 2015 Sep;3:e1273.
12. Curcin V, Ghanem M. Scientific workflow systems - can one size fit all? In: 2008 Cairo International Biomedical Engineering Conference; 2008. p. 1–9.
13. [PDF]A Survey of Data-Intensive Scientific Workflow Management - Inria;.
14. Yarkoni T, Poldrack RA, Nichols TE, Van Essen DC, Wager TD. Large-scale automated synthesis of human functional neuroimaging data. Nat Methods 2011 Aug;8(8):665–670.
15. strace(1): trace system calls/signals - Linux man page;. Accessed: 2017-9-11. https://linux.die.net/man/1/strace.
16. Beaulieu-Jones BK, Greene CS. Reproducible Computational Workflows with Continuous Analysis; 2016.
17. Understanding the GitHub Flow;. Accessed: 2017-1-26. https://guides.github.com/introduction/flow/.
18. Gorgolewski KJ, Auer T, Calhoun VD, Craddock RC, Das S, Duff EP, et al. The brain imaging data structure, a format for organizing and describing outputs of neuroimaging experiments. Sci Data 2016 Jun;3:160044.
19. time(1) - Linux manual page;. Accessed: 2017-1-26. http://man7.org/linux/man-pages/man1/time.1.html.
20. Slurm Workload Manager;. Accessed: 2016-12-6. https://slurm.schedmd.com/plugins.html.
21. SGE Manual Pages;. Accessed: 2015-11-4. http://gridscheduler.sourceforge.net/htmlman/manuals.html.
22. Mojarro A, Hachey J, Ruvkun G, Zuber MT, Carr CE. CarrierSeq: a sequence analysis workflow for low-input nanopore sequencing; 2017.
23. contributors W, JSON; 2015. Accessed: 2015-11-24. https://en.wikipedia.org/w/index.php?title=JSON&oldid=692109528.
24. Stodden V. Reproducibility in Computational and Experimental Mathematics;.
25. Myers K. At the Boundary of Workflows and AI. AAAI Technical Report;.

Figure 1

Click here to download Figure read_calls_octave.png

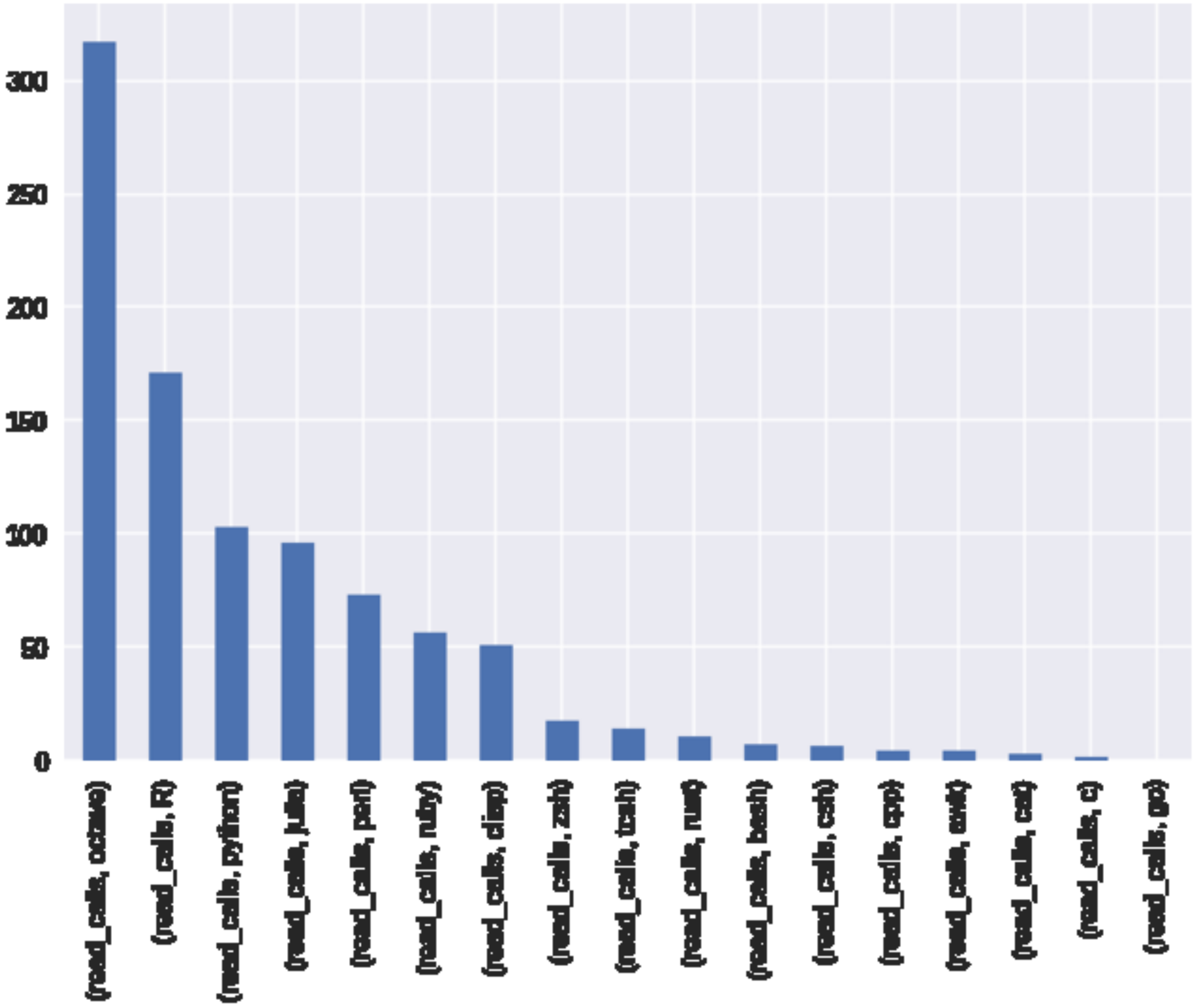

Overleaf LaTeX Manuscript Text

Click here to access/download
Supplementary Material
main.tex

Overleaf LaTeX Manuscript References

Click here to access/download
Supplementary Material
paper-refs.bib

Personal Cover
Click here to download Personal Cover
gigascience_coverletter.pdf

Stanford University

VANESSA SOCHAT
Research Computing

October 31, 2017

Dear Editor,

We are pleased to submit our manuscript titled "The Standard Container Integration Format (SCI-F)" for your consideration as a paper in GigaScience.

The need for reproducible science, and specifically easy to use tools to support it, has been a recent and dire need for scientific and HPC communities. While Linux container technology has done a great deal to capture software dependencies for an analysis, scientific containers are not created in a way to make their contents easily discoverable, and thus usable. The Standard Container Integration Format addresses this need by making it easy for scientists to create and use modular, programatically parseable, and predictably organized scientific containers.

In this paper, we describe the organizational format, and show how it supports reproducible science, and easy generation of containers with multiple entrypoints, each with a modular environment, metadata, and installation steps. We use SCI-F to evaluate container software, provide metrics, serve scientific workflows, and execute a primary function under different contexts. To encourage collaboration and sharing, we provide the implemented standard with version 2.4 of the Singularity software, along with automatic discovery of SCI-F modules via the primary Singularity container registry (https://singularity-hub.org), and a community infrastructure for contributing to SCI-F (https://containers-ftw.github.io/apps).

Importantly, this work was developed after much thinking and feedback from the larger community, and we are excited to share this with GigaScience readers to encourage use of SCI-F to improve reproducibility of scientific containers.

Thank you for your kind consideration.

Sincerely,

Vanessa Sochat

Stanford Research Computing Center
Polya Hall, Stanford CA 94305
vsochat@stanford.edu
