# Supplementary material for: The Scientific Filesystem
Source: Gigascience. 2018 Mar 13;7(5):giy023. doi: 10.1093/gigascience/giy023 (PMC5952957; doi:10.1093/gigascience/giy023)
Supplement: Response_to_Reviewer_Comments_Original_Submission.pdf [file giy023_response_to_reviewer_comments_original_submission.pdf]

Author's Response To Reviewer Comments

Response to Reviewers

Reviewer #1:

Reviewer #1: I thought this was a useful contribution, with a few caveats. The article is heavily slanted towards the "Singularity" container format, and I felt that it would have been possible to be a bit broader in outlook, and include some real "docker" examples, which do not rely on Singularity at all. It could be possible, for example to include SCI-F in a docker container without using Singularity, and to provide an executable in that docker container which could perform all the tasks that the "singularity apps" command provides.

This particular comment was especially impactful, because it is an incredible insight that SCIF doesn't have to be narrowly focused to one container technology (Singularity) or even to containers at all. In response to this feedback, we have made changes to SCIF to make it more broader in outlook as the reviewer suggested. Specifically, we have:

Renamed the organizational format to the Scientific Filesystem (SCIF) in order to not be container-specific. This name is simpler, and reflects the specification focus on filesystem organization for scientific applications.
More thoroughly outlined the specification to be about 1) the filesystem organization, 2) environment variables exposed, and 3) functions to interact with both. A third party application can implement the specification natively (as is done with Singularity) or simply install the client. The latter of these two is what allows for installation of SCIF easily in different container technologies or directly on a host.
Released the specification, along with tutorials and examples for using SCIF in these different contexts at https://vsoch.github.io/scif for continued community contribution and discussion of the specification.
Updated examples in the manuscript to perform equivalent functions with both a Docker and Singularity implementation, all of which are available and documented in their respective repositories (also referenced in the paper).

In the "Example Use Cases", you state that the analysis (of the HelloWorld example) is run "without knowing the specific commands for each language". That may be true, but of course, the creator of each container has to know the specific commands. I didn't think this came across clearly enough.

We have changed the sentence to make this point more clear:

While it is necessary to define the applications installed at the point of creation, using SCIF means that a subsequent user can run the evaluation across modules (languages) without any previous knowledge with a simple for loop.

Some more detailed comments about the wording, and some typos:
In the abstract, you say "we demonstrate that SCI-F is useful by implementing and discussing several use cases, and releasing SCI-F with Singularity..."
I think this should be rephrased, as releasing software does not necessarily demonstrate its usefulness.

Thank you for this point (and the sentiment is mutual!) The sentence is changed to be more geared to reference what the manuscript discusses (beyond just an implementation):

Finally, we demonstrate that SCIF is useful by implementing and discussing several use cases that improve user interaction and understanding of scientific applications.

On page 2, line 12, "weary" should be "wary".
On page 11, line 48, "use cases, are good example" should be "good examples"

Fixed!

The idea of providing some standard data and a user interface/encapsulation for containers is a good one, and well explained in this article.

Thank you!

Reviewer #2

This paper presents a format for describing and interfacing possibly multiple functionalities provided by a single (Singularity) container. Such development is necessary because on one hand although containers typically internally house possibly hundreds of applications, they are exposing only a single one; and on the other hand, even that single application typically is not clearly described to the user, making a container often indeed a black box. Current version of Singularity, through SCI-F (the specification/format presented in the paper), helps to alleviate those problems by providing a unified user entry interfaces to gain insights into the functionalities provided by the container, and to interface them in a consistent matter. This approach opens a number of use-cases (inspection, assistance in scheduling on PBS systems etc) which are presented in detail in this paper.

Before providing any critic and/or recommendations I would like first to thank the author (and other members of the Singularity team) for all the work on Singularity and SingularityHub. Singularity ecosystem is growing and provides a really valuable addition to the toolbelt of any contemporary scientist. SCI-F is also a great step forward and is of great utility.

This is so lovely and kind, thank you! :O)

But, in my opinion, for SCI-F to strive it needs more work and formalization, and this paper needs some revisions to more adequately present SCI-F as a player in the larger ecosystem of

scientific computing and standards.

This author strongly agrees with this sentiment. The vision for SCIF to play a larger role in the ecosystem of scientific computing and standards is spot on, and we have made significant changes to both the formalization of the specification and to the generalizability of scientific filesystems across containers and hosts. We will briefly review the changes we made to address these points, and direct Reviewer #2 to the answer on the first comment of Reviewer #1.

SCIF is renamed to Scientific Filesystem to better reflect a filesystem organization for scientific applications
The SCIF specification is more formally defined to describe a filesystem organization, environment variables, and functions to interact with both.
Documentation, tutorials, and examples are provided across container technologies.

Review

The non-standard "Standard"

Manuscript claims SCI-F to be a standard, not only by using "Standard" in its acronym (which is Ok I guess), but by explicitly referring to it as a "standard" throughout the manuscript.

This author again agrees with the sentiment of the reviewer. A standard requires a high degree of formalization and a community agreement, and so we have more clearly made the distinction in the paper, and scoped SCIF to be a formal specification for such a specification that can enable (or be a strong start to conversation for) a standard.

Unfortunately, I think that SCI-F is not yet in a position to be called a standard, neither a formal one nor a de-facto, even though it could become such. E.g., for SCI-F to become a standard, it should first become container backend-agnostic, so it could be implemented/supported by various container solutions. But ATM it mixes-in specification of within container layout (/scif hierarchy) with tool-specific Singularity file %definitions (%applabels, %apptest, etc); it relies on environment labels which are again tool specific ($SINGULARITY_APPS, etc), which could have been agnostic of the container backend (e.g., by using %SCIF_ prefix instead).

In response to comments and feedback that SCIF does not need to be Singularity (or even container focused) it is in case now both container (and general host) agnostic. With these changes, SCIF is general (and not specific to Singularity containers) and does not include variables specific to Singularity (see https://vsoch.github.io/scif/specification#environment-namespace), and the native implementation in the Singularity software has also been changed (see merged Pull Request to Singularity https://github.com/singularityware/singularity/pull/1238). The definitions themselves (e.g., %appinstall foo) although implemented in Singularity first, were created specifically for SCIF by the lead author. The scattered use of “standard” in the manuscript has been replaced with better descriptors such as “specification” (noun) or “consistent” (adjective).

Typically, standards are embraced either by some consortium (e.g., W3C) or a working group (e.g., how it was done or NIfTI or BIDS in neuroimaging). From the manuscript it is not clear if it is not a "one man standard" (no pun intended).

This was an oversight due to inexperience by the main author, and detail can be provided to justify not being a “one man standard.” The decision of a specification to become a formal standard is largely dependent on the uptake by the broader community, a goal of SCIF and rationale behind the strong community driven effort thus far. The formalized specification detailed in this work is the backbone for a proposed standard, and is developed openly, and open for contribution and feedback at every step. The community involvement thus far includes early discussion on various chat platforms, in-person conversation, discussion on Github pull request (example here https://github.com/singularityware/singularity/pull/826) and issue boards. The first drafts were announced over the Singularity containers list, along with Twitter, and open for collaborative editing as a Google Document. Contributors were encouraged to add their names to the list of authors. Care has been taken in the updated manuscript to remove the term “standard” from the manuscript, and to enable and show a more general use of SCIF over a larger set of scientific software environments.

Singularity could well be the first tool implementing/supporting the "standard" but a Standard should not be tool specific, otherwise we could call any tool's API a "standard" one.

Agreed! With the updates to this manuscript, this is now the case.

Also, ideally, a Standard then should be described in detail in a purely technical specification which would be, again, tool-agnostic. It should describe unambiguously each aspect (ideally in a set of numbered sections to ease referencing), and be versioned with some kind of a "ChangeLog". http://containers-ftw.org/SCI-F/structure.html is indeed a good start for it, but at the moment it is buried within the entirety of http://containers-ftw.org/SCI-F/ which provides similar to this manuscript (nothing wrong with that I guess) overview of SCI-F.

Again, complete agreement! The formal specification is now versioned, and the entry point for the different versions is here: https://sci-f.github.io/scif/specification

from the markdown files here:

https://github.com/vsoch/scif/blob/master/docs/spec

and a CHANGELOG added to track version changes and releases https://github.com/sci-f/sci-f.github.io/blob/master/CHANGELOG.md.

Overall, I would recommend the author(s) to "tone down" the claim on establishing a Standard, and state that this Format could potentially be generalized to become a Standard and to invite interested parties to participate/contribute in such formalization.

We agree, and have largely removed the term “standard” from the manuscript and added language to encourage further contribution and participation.

Comparison to other solutions

Reading the manuscript gives a wrong perception of SCI-F to be the solution which all of a sudden was developed to finally bring modularity and structure into computing, when before it was just a black box darkness and chaos of tools. I think it is a bit misleading presentation, primarily because of not mentioning or clearly comparing to existing solutions across a variety of levels.

This was an oversight, and we've added a section about other Module Solutions to the manuscript and added more references to cite other tools. The author is aware that the background is extensive, and the brief mention is due to the understanding that the interested reader has knowledge about the enormous number of options and efforts, all highly reputable.

SCIF remains unique to any single effort in isolation, however. While different components of SCIF have been done in other software (e.g., environment modules with lmod) SCIF is unique in that SCIF couples together features of defining not just an environment, but also metadata, help documentation, entry points, tests, and files. SCIF can be used in conjunction with these tools, and isn't intended to replace them.

In the context of a container, the most likely place for a SCIF to be installed, we believe the analogy of a black box to be spot on. The way containers (and hosts that a researcher might shell into like on a cluster) are designed is to be mostly reliant on (external) documentation or knowledge for usage. Without working on a standard like SCIF the inability to understand and introspect scientific applications will remain challenging.

SCI-F is not really specific to containers...

At large, SCI-F itself has little (if nothing at all) to do with containers! SCI-F suggests 1. /scif directories/files hierarchy, 2. (ATM Singularity specific) documentation/"build"/execution specification (Singularity file %definitions), 3. (implemented in Singularity) user interface to access the "apps".

So, it could be easily implemented on a "bare system" and thus also by any container solution. So, in principle, a system (or container) administrator (or creator) could provide a list of apps accessible through some `scif` command line interface. And that opens presentation of SCI-F into a much larger universe of computing environments, where the same problems existed for decades and for which solutions actually WERE provided: various distributions (Linux distributions, conda, etc), environment-modules (http://modules.sourceforge.net), etc.

We are very glad for this feedback from the reviewer, because it inspired a few more months of work to better solidify the specification, create a client, and show this kind of usage across different hosts and container technologies.

They all already provide functionality to not only build, install ("pull" in container's world), but also to inspect, and to search (from distribution specific to "man -k" for any *nix system) for needed "apps".

These tools are great, and we can give more attention to the other ecosystems working within the realm of reproducible scientific software. Every effort is laudable, and they do not necessarily need to be mutually exclusive. Many are more specialized for package management, and less optimized for building a filesystem that is predictable and programatically parseable. It is important to reveal metadata (inspect) and find a package that exists (search), however this is just a shared component between package (and module) managers and SCIF, which includes a specification for the organization and interaction with the content itself. The efforts have synergy, and this is more a reflection of a common necessity.

This author agrees that there are very often many different initiatives that aim to work on solving similar problems. This is true across much time, and for tools and software outside of the scope of science. However until something “sticks” or “takes off,” there is always potential for doing better.

Quite often they are actually guided by standards (such as the mentioned FHS) or clear specifications, API
and/or support tools. They do allow for comparison across any environments, including containers (see e.g.
https://github.com/GoogleCloudPlatform/container-diff), thus somewhat contradicting the stated issue "Containers are not consistent to allow for comparison across containers...", "... contents are not programmatically understandable", "container internal infrastructure is not modular").

The statement “Containers are not consistent” is more correctly “container contents are not consistent,” and we have adjusted this line to be more clear. Also note that these statements are no longer directed at containers.

But SCI-F is presented without any mentioning or comparison to existing solutions for modular systems design as if none of those exists or applies.

As stated previously, we have adjusted this language, and added how the container-diff could be used with SCIF to enhance it. Note that the container-diff repository was published after the paper was written, and still is limited to a particular set of container technologies. It also assumes wanting to do more of a global comparison (container A vs container B), which doesn’t give much insight to how some scientific (or other) application in the container is distinguished from the base operating system. Adding SCIF here would allow you to ascribe meaning and groupings to the content of containers before doing this. SCIF can be used with such a tool and can only enhances the comparison.

I do understand that authors aim to describe a unified "user interface" to the containerized applications. SCI-F, as applied to (Singularity) containers, fills the gap between those very rich and non-specialized environments (which are BTW typically are quite modular, and are pre-installed inside those Singularity containers) and typical containers which have only a single

point of entry, and where even that one is usually (but not necessarily) not properly documented thus indeed making many containers into black boxes.

Given the change that SCIF is now separate from Singularity, and shown to work with one entrypoint (Docker), the manuscript is setting up the base for a unified user-interface to programmatic reproducibility, given using SCIF in context of a reproducible container. This is a compelling goal for the continued development of SCIF.

I think that the manuscript should position SCI-F not in isolation from those (I list some other below as well) other solutions, but rather to relate to them, along the lines of "SCI-F provides a target, use-case specific interface to the (possibly richer) environment".

Agreed! Please see the section on other solutions for Modularity where we have discussed this, along with mentioning the container-diff and other resources that you mentioned.

"Gateway" command line interfaces

I think it might be worth relating to a growing in adoption paradigm for command line interfaces -- a "gateway" interface where the main command provides description/interface to sub-commands (e.g., as in docker, singularity, git, ...). Here, `scif` could in principle (if was indeed standardized) provide a similar common interface to the apps in any environment, or exposed by container manager (such as Singularity).

Valid points and now included. See the “Client Requirements” section of the specification where this interface is now defined.

https://sci-f.github.io/spec-v1#client-requirements

Also, since the manuscript is offering a new solution, it might be worth directly outlining the benefit(s) of dedicated client-side commands (apps, run --app) in comparison to e.g. establishing similar gateway command for the entire container and relying on the invocation (--help, app --help) -- the interface to the majority of command line commands users already aware of, and a user interface design principle they are familiar with.

Both SCIF and Singularity offer the same familiar gateway that is easily discovered to see help for the entire software (e.g., singularity help or scif help) along with a component of it (scif help). Now, given that a SCIF is installed in a Singularity container (as it could be on a host or Docker container) if the user executes the container (or scif) entrypoint with /entrypoint help the expected behavior results. The original --app help components of Singularity are another means to show these help commands that are provided natively by the Singularity software, provided for Singularity users that are more familiar with the Singularity command gateway that uses arguments flags (e.g. --app). importantly, SCIF was developed and integrated into Singularity, and not the other way around), and there are no strict requirements that a native integration with a SCIF filesystem organization cannot provide additional means to interact with it that are more

appropriate for the tool in question.

Other container-based interfaces

A comparison to other container-based solutions which provide clear specifications for the invocation and possibly even documentation (--help), such as BIDS-Apps (http://bids-apps.neuroimaging.io) is also missing.

There are many container-based solutions that have an entrypoint with help, and in the case of BIDS it is commonly a Python entrypoint that uses argparse. In that SCIF is not a container solution exclusively, it would be hardly possible and not the goal of the paper to list them all! We point out, as we mention in the paper, that writing these entrypoints requires some knowledge of Python, white writing a SCIF entrypoint means just writing a block of text and having confidence it will be reachable. In the case of using something like argparse in Python, the implementation (in using Python) requires initializing many of the required modules. This would mean that if there is an error to importing something, the help would be unreachable. This has been in fact an issue with some of the BIDS containers that are converted from one format to another (Docker to Singularity). In that BIDS installs content to root's home, and there are whiteout files present with higher level permissions than the user has, many of these containers when converted from Docker cannot finish import or even show help documentation. The help is unreachable. SCIF solves this issue by clearly separating the two things. If the application is somehow broken, the user can still look at the help.

Although the manuscript tries to explicitly differentiate itself from "workflow managers", principles of some workflow manager container-based solutions (e.g., Gears of Flywheel, boutiques) fall exactly into the domain which SCI-F tries to cover: description of the available app(s), location of its inputs/outputs (to be mounted, etc) -- all those aspects are typically one way (standardized paths) or another (command line options) are formalized in the aforementioned solutions.

There is overlap in ideas and principles, and this is ok. There is demand for various solutions and focus points, and some of the most interesting use cases are when a tool is discovered to be useful beyond its initial anticipated design. Some could build a basic workflow manager on SCIF for very simple use cases (e.g., see the carrierseq example https://github.com/vsoch/carrierseq/blob/master/carrierseq_singularity.sh) It cannot be forgotten that SCIF is also a file system organization, set of environment variables, and definition of interactions with it, which has different, though sometimes overlapping, goals than something like a json file to define general inputs and outputs for execution of a pipeline.

Once again, readers would benefit from at least a brief and pragmatic comparison to those other existing solutions, with reasoning for the benefit from multiple apps being exposed from a single container as compared to multiple containers with the same base, just different interfaced apps (disk-space efficiently achieved with docker due to overlays). Here you could also relate to the use-cases (inspection, testing) you present later in the manuscript.

So, overall, I would recommend to adjust Introduction and Goals to relate better to existing

solutions and present SCI-F a little bit more to the point. It is clear that adding "apps" support to Singularity containers interface is an important addition. Either it would remain a Singularity specific Format, or to become anyhow a Standard and/or would be adopted by other existing frameworks is to be seen.

Done! I hope the (substantial, took me months of work to do) changes have resolved these concerns.

Unclear/unused relationship with singularity-hub.org

The cover letter talks about "automatic discovery of SCI-F modules via the ... singularity-hub.org". The manuscript (p.12 lines 19-26) only mentions that the Hub provides a build service, and I have not found any example/explanation on how "automated discovery" should be accomplished. I have also failed to find/pull those sample apps (such as the ones corresponding to cseq.simg).

The automatic discovery of SCIF modules in a container comes by way of just running the entrypoint with “apps,” as is shown in the manuscript, to discover what is installed without having built the container. This simple command would allow any researcher wanting to do an analysis based on comparing a set of SCIF apps in some way to discover them. Singularity Hub is a simple example of that - the container is built, and then apps are discovered (along with metadata) to render for search/browsability on the site automatically. For example, here are containers that have been discovered to have an app called “RScript.”

https://singularity-hub.org/apps/Rscript

So Singularity Hub simply finds app sections in a recipe, and makes it possible to search containers based on them. There are no extra or special containers built, beyond what the user has provided.

I would recommend to make examples more complete/easily reproducible by providing for each new app/image a "singularity pull" command invocation to get that app/image so readers could try. At the moment, text just references to github repositories, hinting that reader should build those apps on their own? Note that it might hinder reproducibility (apps/environments are not necessarily 100% identically re-buildable -- base system might provide updates, and also see e.g., https://wiki.debian.org/ReproducibleBuilds)

You got it! The Docker Hub containers are also provided, and likely will be an easier entrypoint (more users are familiar / use Docker).

Modularity

(Virtually) all containers are internally modular

"A container with distinct, predictable locations for software

modules and data is modular" could be answered here if we consider FHS (which is not referred to): /usr/bin/NAME and /usr/share/data/NAME -- as modular as it gets. Why SCI-F is not using this part of the standard? (the answer is somewhat given in the next statement)

We do mention FHS in the background that explains the initial rationale for developing SCIF. The general idea is that a SCIF must be distinguished from the base operating system. There are likely to be libraries shared with a host and a scientific application, but the core of the scientific software should be easy to untangle from it. Having the base /scif/apps makes a confident statement that "The software found here is for the scientific applications." If we install scientific apps to /usr/bin and then come back at a later time, we would confuse the apps with other software that has been installed in /usr/bin.

This internal modularity is somewhat hinted on while talking about "Development" kind of modularity. Making this gradient even more explicit by noting that majority of containers internally are quite modular already, but not "specialized", might satisfy such readers as yours truly.

This is a really good idea - there is perhaps a distinction to make between "technology-oriented modularity" and "scientist-oriented modularity." We have adjusted this section to mention this.

Modularity is not a panacea for "mobility"

"Further, this structure allows for easy movement of modules between containers. If a module carries with it complete information about installation and dependencies, it could be easily be installed in another container."

First of all it is not exactly clear either authors by "module" refer to an installed app (/scif/apps/foo, let's call it a binary distribution) as hinted by the beginning of that paragraph, or an app %specification within Singularity file (let's call it a "source distribution").

Again, problem is not exactly a new one if you refer back to Linux distributions. They are modular, provide clear specifications, build and installation success guarantees, even recently build reproducibility (https://wiki.debian.org/ReproducibleBuilds) etc. You can install the modules you want, thus achieving to some degree "move" and "clone" capabilities. But,

But moving across environments or containers, both binary or even build-from-source forms might be not that trivial for any non-trivial case: packaging tools and package names would differ across distributions, names of dependencies might change between releases, etc. I am not even talking about binary compatibility, unless app is built with that in mind bundling all dynamic libraries or compiling with static linkage. My point is -- "modularity" is not a panacea here, and a promise of being easily move "modules" between containers comes not without limitations. The manuscript should be adjusted to reflect situation more realistically.

Thank you for your thoughtful comments. We had a hard time describing and defining modularity, indeed, and for this reason had several different "kinds" of modularity, depending on

the usage. We have adjusted the manuscript to make it clear that SCIF != reproducibility, but that it greatly enhances reproducibility for a container. We also pointed out that the same checks and concerns would still apply with installing an equivalent module across different operating systems. SCIF can define apps, but it still requires another set of eyes (a human being) to put them into a recipe (or on a host) with a particular operating system, combined with other things, and ensure this is a good idea (see Limitations).

Integrations and Tools

Container Bases

Again, no hint on comparison to other existing solutions or possible limited "mobility" of the apps. On the other hand though, similar in idea solutions also could be noted (e.g. see https://github.com/kaczmarj/neurodocker).

Existing solutions on mobility and packaging have been added, and a citation to the referenced above added.

Container Assessment

Again, an analogy between container/app and a regular software/tool could be made. Annotation and assessment of those is nothing new, various approaches exist to provide an identity (e.g., RRID), grouping (e.g. blends in Debian), functional assessment (all the helpers like time, etc), etc etc. No connection anyhow is made to existing ecosystem of those and only quite a lengthy exploration of possible assessments is presented, with its length, in my opinion, distracting from actual presentation of SCI-F. I would advise on shortening this section, making clearer analogies with how software typically is annotated, and how SCI-F could be helpful.

Thank you for this feedback! Changes have been made to make it clear that the principle also works outside of containers. These specific sections are intended are also intended as abstract examples to inspire the reader's thinking, and are not concrete comparisons to existing tools.

Modular Metrics Evaluation

I wonder if this use-case doesn't actually undermine the "modularity" of the SCI-F approach? Whenever in the case of regular apps, I could easily combine a metric (e.g. "time") with any app (e.g. "helloworld" or "byeworld"), SCI-F seems to suggest create metrics dedicated to the only "app" provided by the container and there is no way to apply a metric to another app within the same container. The same is applicable to "Contextual Running".

SCIF would allow for the use cases mentioned by the reviewer (running an app across some single application in the container) or being able to run an application against any other application in the container, given that the application is written to take another application name

as a command line argument (the metrics example now demonstrates this). Generally, the exact implementation (and how a metrics SCIF could work) is up to the user.

Moreover, I do not really see people eager to provision those "metrics" for their SCI-F containers. Similar to contextual running. I wondered, if things are "standardized" really, could there be a library of metrics, contextual runners, which could be automagically provisioned by the SCI-F platform to be then invoked for any app within a container (with API like "singularity run --app time --app helloworld metrics.img" or alike)? That would be a really valuable addition provided by SCI-F. But since not to happen for this publication/version, I just wanted to express my opinion and not sure what steps could/should be taken to improve the manuscript in this regard.

This is something likely of interest to administrators (and not users), and would be very feasible using SCIF as the basis to install and use metrics. As for generation of some kind of metrics database, this author most definitely would enjoy thinking about this task once the specification is properly disseminated. The examples provided in the manuscript do not intend to be formal metrics, but rather just examples.

Future Work items could be more pragmatic

Mapping of container landscape

Have I missed somewhere a "call home" feature of singularity to even collect the information needed for such analysis?
I you are interested in such analyses, I would recommend to look at an existing collection of "co-installed" apps, as e.g. could be provided by Debian popularity contest. Anyways, I do not see SCI-F really providing some kind of ultimate unique backbone technology to achieve desired.

These discussions are meant to inspire this kind of work as “next steps.” This is a fantastic idea, and would be possible using SCIF.

Artificial Intelligence (AI) Generated Containers

This section reads as a total SCI-Fi, not much different from hopes for Lisp to "solve" AI by mid-80s. In addition, in my opinion, SCI-F provides no additional benefit to achieve this specific goal in comparison to e.g. taking an existing rich environment with thousands of apps (e.g., Debian or conda) and a collection of (prescribed even in greater detail, as e.g. in nipype; or Flywheel gears or boutiques) interfaces to the scientific apps. Neither SCI-F anyhow touches upon management of the collection(s) of SCI-F'ized containers, which would be needed to aggregate and manage information across such collections of apps for any kind of composition.

This is also just a section to inspire creativity, because sometimes that kind of general brainstorming can trigger a really different and interesting idea. Papers are commonly boring, and especially one about a specification, these sections are very short and help to balance it.

SCIF opens up an abstraction from underlying programming languages and packaging logic to work with scientific applications, and this alone opens up new opportunities.

I do not want to (and can't ;)) diminish awesomeness of Singularity, SingularityHub, and now SCI-F but overall, I would advise to include more pragmatic and thus better resonating with the reader possible future work items.
Here are some ideas which came to my mind:

- refactoring SCI-F into a proper Standard
- so it could be adopted by other containerized (or not) solutions
- include the supported Format version to be reported by the containers
(the Format/Standard will evolve, so you will find it needed at some point)
- allowing for better modularity (e.g., metrics to be applied to any of the apps)
- further enhancement to the interface
- `apps` to provide a brief description for each app (compare to e.g. `git --help`), not just names
- `apps` to provide grouping of commands (apps vs metrics etc, again e.g. `git --help` to compare against)

All of these are done! The only command that isn't changed is apps, as it's common to pipe this into a script to be used to discover and reference the app.

- Wishlist items:
- container (from shub:// at least) might like to provide information on where it could be obtained from and include that information in `inspect` output (see my above comment on inability to figure out where to get those apps)
- Singularity could provide command line interface to search for images/apps e.g. on shub:// .

Although the Singularity Hub API and interface to it are separate from SCIF, sometimes wishes do come true :)

https://singularityhub.github.io/sregistry-cli/client-hub#search
