# Supplementary material for: The Scientific Filesystem
Source: Gigascience. 2018 Mar 13;7(5):giy023. doi: 10.1093/gigascience/giy023 (PMC5952957; doi:10.1093/gigascience/giy023)
Supplement: Response_to_Reviewer_Comments_Revision_1.pdf [file giy023_response_to_reviewer_comments_revision_1.pdf]

Author's Response To Reviewer Comments

Dear Editor,

The following changes have been made to the manuscript:

- The DOI in the GigaDB has been added and cited as reference 37, and is mentioned in the "Availability of supporting data."
- SCIF has been registered in the SciCrunch.org database, with RRID RRID:SCR_016105. The RRID, along with other tools' RRID, has been added next to the software abbreviation list for ease of discoverability.
- The Abbreviations section has been added to the appendix in alphabetical order.
- Docker and Singularity have been added to the abstract to make it more clear that SCIF can help with reproducible containers.

Thank you!

Best,

Vanessa
