# Supplementary material for: The Scientific Filesystem
Source: Gigascience. 2018 Mar 13;7(5):giy023. doi: 10.1093/gigascience/giy023 (PMC5952957; doi:10.1093/gigascience/giy023)
Supplement: Reviewer_1_Report_(Original_Submission) - Chris Richardson, PhD — 20 Nov 2017 Reviewed [file giy023_reviewer_1_report_(original_submission).pdf]

Reviewer Report

Title: The Scientific Filesystem (SCIF)

Version: Original Submission **Date:** 20 Nov 2017

Reviewer name: Chris Richardson, PhD

Reviewer Comments to Author:

I thought this was a useful contribution, with a few caveats. The article is heavily slanted towards the "Singularity" container format, and I felt that it would have been possible to be a bit broader in outlook, and include some real "docker" examples, which do not rely on Singularity at all. It could be possible, for example to include SCI-F in a docker container without using Singularity, and to provide an executable in that docker container which could perform all the tasks that the "singularity apps" command provides.

In the "Example Use Cases", you state that the analysis (of the HelloWorld example) is run "without knowing the specific commands for each language". That may be true, but of course, the creator of each container has to know the specific commands. I didn't think this came across clearly enough.

Some more detailed comments about the wording, and some typos:
In the abstract, you say "we demonstrate that SCI-F is useful by implementing and discussing several use cases, and releasing SCI-F with Singularity..."
I think this should be rephrased, as releasing software does not necessarily demonstrate its usefulness.
On page 2, line 12, "weary" should be "wary".
On page 11, line 48, "use cases, are good example" should be "good examples"

The idea of providing some standard data and a user interface/encapsulation for containers is a good one, and well explained in this article.

Level of Interest

Please indicate how interesting you found the manuscript: An article of importance in its field

Quality of Written English

Please indicate the quality of language in the manuscript: Acceptable

Declaration of Competing Interests

Please complete a declaration of competing interests, considering the following questions:

- Have you in the past five years received reimbursements, fees, funding, or salary from an organisation that may in any way gain or lose financially from the publication of this manuscript, either now or in the future?
- Do you hold any stocks or shares in an organisation that may in any way gain or lose financially from the publication of this manuscript, either now or in the future?
- Do you hold or are you currently applying for any patents relating to the content of the manuscript?
- Have you received reimbursements, fees, funding, or salary from an organization that holds or has applied for patents relating to the content of the manuscript?
- Do you have any other financial competing interests?
- Do you have any non-financial competing interests in relation to this paper?

If you can answer no to all of the above, write 'I declare that I have no competing interests' below. If your reply is yes to any, please give details below.

I declare that I have no competing interests

I agree to the open peer review policy of the journal. I understand that my name will be included on my report to the authors and, if the manuscript is accepted for publication, my named report including any attachments I upload will be posted on the website along with the authors' responses. I agree for my report to be made available under an Open Access Creative Commons CC-BY license (http://creativecommons.org/licenses/by/4.0/). I understand that any comments which I do not wish to be included in my named report can be included as confidential comments to the editors, which will not be published.

I agree to the open peer review policy of the journal

To further support our reviewers, we have joined with Publons, where you can gain additional credit to further highlight your hard work (see: https://publons.com/journal/530/gigascience). On publication of this paper, your review will be automatically added to Publons, you can then choose whether or not to claim your Publons credit. I understand this statement.

Yes
