# Supplementary material for: The Scientific Filesystem
Source: Gigascience. 2018 Mar 13;7(5):giy023. doi: 10.1093/gigascience/giy023 (PMC5952957; doi:10.1093/gigascience/giy023)
Supplement: Reviewer_2_Report_(Original_Submission) -- Yaroslav Halchenko — 12 Dec 2017 Reviewed [file giy023_reviewer_2_report_(original_submission).pdf]

Reviewer Report

Title: The Scientific Filesystem (SCIF)

Version: Original Submission **Date:** 12 Dec 2017

Reviewer name: Yaroslav Halchenko

Reviewer Comments to Author:

Brief summary

This paper presents a format for describing and interfacing possibly multiple functionalities provided by a single (Singularity) container. Such development is necessary because on one hand although containers typically internally house possibly hundreds of applications, they are exposing only a single one; and on the other hand, even that single application typically is not clearly described to the user, making a container often indeed a black box. Current version of Singularity, through SCI-F (the specification/format presented in the paper), helps to alleviate those problems by providing a unified user entry interfaces to gain insights into the functionalities provided by the container, and to interface them in a consistent matter. This approach opens a number of use-cases (inspection, assistance in scheduling on PBS systems etc) which are presented in detail in this paper.

Before providing any critic and/or recommendations I would like first to thank the author (and other members of the Singularity team) for all the work on Singularity and SingularityHub. Singularity ecosystem is growing and provides a really valuable addition to the toolbelt of any contemporary scientist. SCI-F is also a great step forward and is of great utility. But, in my opinion, for SCI-F to strive it needs more work and formalization, and this paper needs some revisions to more adequately present SCI-F as a player in the larger ecosystem of scientific computing and standards.

Review

The non-standard "Standard"

Manuscript claims SCI-F to be a standard, not only by using "Standard" in its acronym (which is Ok I guess), but by explicitly referring to it as a "standard" throughout the manuscript.

Unfortunately, I think that SCI-F is not yet in a position to be called a standard, neither a formal one nor a de-facto, even though it could become such. E.g., for SCI-F to become a standard, it should first become container backend-agnostic, so it could be implemented/supported by various container solutions. But ATM it mixes-in specification of within container layout (/scif hierarchy) with tool-specific Singularity file %definitions (%applabels, %apptest, etc); it relies on environment labels which are again tool specific ($SINGULARITY_APPS, etc), which could have been agnostic of the container backend (e.g., by using %SCIF_ prefix instead). Typically, standards are embraced either by some consortium (e.g.,

W3C) or a working group (e.g., how it was done or NIfTI or BIDS in neuroimaging). From the manuscript it is not clear if it is not a "one man standard" (no pun intended).

Singularity could well be the first tool implementing/supporting the "standard" but a Standard should not be tool specific, otherwise we could call any tool's API a "standard" one.

Also, ideally, a Standard then should be described in detail in a purely technical specification which would be, again, tool-agnostic. It should describe unambiguously each aspect (ideally in a set of numbered sections to ease referencing), and be versioned with some kind of a "ChangeLog". http://containers-ftw.org/SCI-F/structure.html is indeed a good start for it, but at the moment it is buried within the entirety of http://containers-ftw.org/SCI-F/ which provides similar to this manuscript (nothing wrong with that I guess) overview of SCI-F.

Overall, I would recommend the author(s) to "tone down" the claim on establishing a Standard, and state that this Format could potentially be generalized to become a Standard and to invite interested parties to participate/contribute in such formalization.

Comparison to other solutions

Reading the manuscript gives a wrong perception of SCI-F to be the solution which all of a sudden was developed to finally bring modularity and structure into computing, when before it was just a black box darkness and chaos of tools. I think it is a bit misleading presentation, primarily because of not mentioning or clearly comparing to existing solutions across a variety of levels.

SCI-F is not really specific to containers...

At large, SCI-F itself has little (if nothing at all) to do with containers! SCI-F suggests 1. /scif directories/files hierarchy, 2. (ATM Singularity specific) documentation/"build"/execution specification (Singularity file %definitions), 3. (implemented in Singularity) user interface to access the "apps".

So, it could be easily implemented on a "bare system" and thus also by any container solution. So, in principle, a system (or container) administrator (or creator) could provide a list of apps accessible through some `scif` command line interface. And that opens presentation of SCI-F into a much larger universe of computing environments, where the same problems existed for decades and for which solutions actually WERE provided: various distributions (Linux distributions, conda, etc), environment-modules (http://modules.sourceforge.net), etc. They all already provide functionality to not only build, install ("pull" in container's world), but also to inspect, and to search (from distribution specific to "man -k" for any *nix system) for needed "apps". Quite often they are actually guided by standards (such as the mentioned FHS) or clear specifications, API and/or support tools. They do allow for comparison across any environments, including containers (see e.g. https://github.com/GoogleCloudPlatform/container-diff), thus somewhat contradicting the stated issue "Containers are not consistent to allow for comparison across containers...", "... contents are not

programmatically understandable", "container internal infrastructure is not modular"). But SCI-F is presented without any mentioning or comparison to existing solutions for modular systems design as if none of those exists or applies.

I do understand that authors aim to describe a unified "user interface" to the containerized applications. SCI-F, as applied to (Singularity) containers, fills the gap between those very rich and non-specialized environments (which are BTW typically are quite modular, and are pre-installed inside those Singularity containers) and typical containers which have only a single point of entry, and where even that one is usually (but not necessarily) not properly documented thus indeed making many containers into black boxes.

I think that the manuscript should position SCI-F not in isolation from those (I list some other below as well) other solutions, but rather to relate to them, along the lines of "SCI-F provides a target, use-case specific interface to the (possibly richer) environment".

TODO: compare modularity of those individual apps to the suggested "metrics" use-case which can't be applied to multiple apps in the container (somewhat ruined modularity "promise")

"Gateway" command line interfaces

I think it might be worth relating to a growing in adoption paradigm for command line interfaces -- a "gateway" interface where the main command provides description/interface to sub-commands (e.g., as in docker, singularity, git, ...). Here, `scif` could in principle (if was indeed standardized) provide a similar common interface to the apps in any environment, or exposed by container manager (such as Singularity).

Also, since the manuscript is offering a new solution, it might be worth directly outlining the benefit(s) of dedicated client-side commands (apps, run --app) in comparison to e.g. establishing similar gateway command for the entire container and relying on the invocation (--help, app --help) -- the interface to the majority of command line commands users already aware of, and a user interface design principle they are familiar with.

Other container-based interfaces

A comparison to other container-based solutions which provide clear specifications for the invocation and possibly even documentation (--help), such as BIDS-Apps (http://bids-apps.neuroimaging.io) is also missing. Although the manuscript tries to explicitly differentiate itself from "workflow managers", principles of some workflow manager container-based solutions (e.g., Gears of Flywheel, boutiques) fall exactly into the domain which SCI-F tries to cover: description of the available app(s), location of its inputs/outputs (to be mounted, etc) -- all those aspects are typically one way (standardized paths) or

another (command line options) are formalized in the aforementioned solutions.

Once again, readers would benefit from at least a brief and pragmatic comparison to those other existing solutions, with reasoning for the benefit from multiple apps being exposed from a single container as compared to multiple containers with the same base, just different interfaced apps (disk-space efficiently achieved with docker due to overlays). Here you could also relate to the use-cases (inspection, testing) you present later in the manuscript.

So, overall, I would recommend to adjust Introduction and Goals to relate better to existing solutions and present SCI-F a little bit more to the point. It is clear that adding "apps" support to Singularity containers interface is an important addition. Either it would remain a Singularity specific Format, or to become anyhow a Standard and/or would be adopted by other existing frameworks is to be seen.

Unclear/unused relationship with singularity-hub.org

The cover letter talks about "automatic discovery of SCI-F modules via the ... singularity-hub.org". The manuscript (p.12 lines 19-26) only mentions that the Hub provides a build service, and I have not found any example/explanation on how "automated discovery" should be accomplished. I have also failed to find/pull those sample apps (such as the ones corresponding to cseq.simg).

I would recommend to make examples more complete/easily reproducible by providing for each new app/image a "singularity pull" command invocation to get that app/image so readers could try. At the moment, text just references to github repositories, hinting that reader should build those apps on their own? Note that it might hinder reproducibility (apps/environments are not necessarily 100% identically re-buildable -- base system might provide updates, and also see e.g., https://wiki.debian.org/ReproducibleBuilds)

Modularity

(Virtually) all containers are internally modular

"A container with distinct, predictable locations for software
modules and data is modular" could be answered here if we consider FHS (which is not referred to): /usr/bin/NAME and /usr/share/data/NAME -- as modular as it gets. Why SCI-F is not using this part of the standard? (the answer is somewhat given in the next statement)

This internal modularity is somewhat hinted on while talking about "Development" kind of modularity. Making this gradient even more explicit by noting that majority of containers internally are quite modular already, but not "specialized", might satisfy such readers as yours truly.

Modularity is not a panacea for "mobility"

"Further, this structure allows for easy movement of modules between containers. If a module carries with it complete information about installation and dependencies, it could be easily be installed in another container."

First of all it is not exactly clear either authors by "module" refer to an installed app (/scif/apps/foo, let's call it a binary distribution) as hinted by the beginning of that paragraph, or an app %specification within Singularity file (let's call it a "source distribution").

Again, problem is not exactly a new one if you refer back to Linux distributions. They are modular, provide clear specifications, build and installation success guarantees, even recently build reproducibility (https://wiki.debian.org/ReproducibleBuilds) etc. You can install the modules you want, thus achieving to some degree "move" and "clone" capabilities. But,

But moving across environments or containers, both binary or even build-from-source forms might be not that trivial for any non-trivial case: packaging tools and package names would differ across distributions, names of dependencies might change between releases, etc. I am not even talking about binary compatibility, unless app is built with that in mind bundling all dynamic libraries or compiling with static linkage. My point is -- "modularity" is not a panacea here, and a promise of being easily move "modules" between containers comes not without limitations. The manuscript should be adjusted to reflect situation more realistically.

Integrations and Tools

Container Bases

Again, no hint on comparison to other existing solutions or possible limited "mobility" of the apps. On the other hand though, similar in idea solutions also could be noted (e.g. see https://github.com/kaczmarj/neurodocker).

Container Assessment

Again, an analogy between container/app and a regular software/tool could be made. Annotation and assessment of those is nothing new, various approaches exist to provide an identity (e.g., RRID), grouping (e.g. blends in Debian), functional assessment (all the helpers like time, etc), etc etc. No connection anyhow is made to existing ecosystem of those and only quite a lengthy exploration of possible assessments is presented, with its length, in my opinion, distracting from actual presentation of SCI-F. I would advise on shortening this section, making clearer analogies with how software typically is annotated, and how SCI-F could be helpful.

Modular Metrics Evaluation

I wonder if this use-case doesn't actually undermine the "modularity" of the SCI-F approach? Whenever

in the case of regular apps, I could easily combine a metric (e.g. "time") with any app (e.g. "helloworld" or "byeworld"), SCI-F seems to suggest create metrics dedicated to the only "app" provided by the container and there is no way to apply a metric to another app within the same container. The same is applicable to "Contextual Running".

Moreover, I do not really see people eager to provision those "metrics" for their SCI-F containers. Similar to contextual running. I wondered, if things are "standardized" really, could there be a library of metrics, contextual runners, which could be automagically provisioned by the SCI-F platform to be then invoked for any app within a container (with API like "singularity run --app time --app helloworld metrics.img" or alike)? That would be a really valuable addition provided by SCI-F. But since not to happen for this publication/version, I just wanted to express my opinion and not sure what steps could/should be taken to improve the manuscript in this regard.

Future Work items could be more pragmatic

Mapping of container landscape

Have I missed somewhere a "call home" feature of singularity to even collect the information needed for such analysis?
I you are interested in such analyses, I would recommend to look at an existing collection of "co-installed" apps, as e.g. could be provided by Debian popularity contest. Anyways, I do not see SCI-F really providing some kind of ultimate unique backbone technology to achieve desired.

Artificial Intelligence (AI) Generated Containers

This section reads as a total SCI-Fi, not much different from hopes for Lisp to "solve" AI by mid-80s. In addition, in my opinion, SCI-F provides no additional benefit to achieve this specific goal in comparison to e.g. taking an existing rich environment with thousands of apps (e.g., Debian or conda) and a collection of (prescribed even in greater detail, as e.g. in nipype; or Flywheel gears or boutiques) interfaces to the scientific apps. Neither SCI-F anyhow touches upon management of the collection(s) of SCI-F'ized containers, which would be needed to aggregate and manage information across such collections of apps for any kind of composition.

I do not want to (and can't ;)) diminish awesomeness of Singularity, SingularityHub, and now SCI-F but overall, I would advise to include more pragmatic and thus better resonating with the reader possible future work items. Here are some ideas which came to my mind:

- refactoring SCI-F into a proper Standard
- so it could be adopted by other containerized (or not) solutions
- include the supported Format version to be reported by the containers
(the Format/Standard will evolve, so you will find it needed at some point)
- allowing for better modularity (e.g., metrics to be applied to any of the apps)

- further enhancement to the interface
- `apps` to provide a brief description for each app (compare to e.g. `git --help`), not just names
- `apps` to provide grouping of commands (apps vs metrics etc, again e.g. `git --help` to compare against)
- Wishlist items:
- container (from shub:// at least) might like to provide information on where it could be obtained from and include that information in `inspect` output (see my above comment on inability to figure out where to get those apps)
- Singularity could provide command line interface to search for images/apps e.g. on shub:// .

Level of Interest

Please indicate how interesting you found the manuscript: An article of importance in its field

Quality of Written English

Please indicate the quality of language in the manuscript: Acceptable

Declaration of Competing Interests

Please complete a declaration of competing interests, considering the following questions:

- Have you in the past five years received reimbursements, fees, funding, or salary from an organisation that may in any way gain or lose financially from the publication of this manuscript, either now or in the future?
- Do you hold any stocks or shares in an organisation that may in any way gain or lose financially from the publication of this manuscript, either now or in the future?
- Do you hold or are you currently applying for any patents relating to the content of the manuscript?
- Have you received reimbursements, fees, funding, or salary from an organization that holds or has applied for patents relating to the content of the manuscript?
- Do you have any other financial competing interests?
- Do you have any non-financial competing interests in relation to this paper?

If you can answer no to all of the above, write 'I declare that I have no competing interests' below. If your reply is yes to any, please give details below.

I declare that I have no competing interests

I agree to the open peer review policy of the journal. I understand that my name will be included on my report to the authors and, if the manuscript is accepted for publication, my named report including any attachments I upload will be posted on the website along with the authors' responses. I agree for my report to be made available under an Open Access Creative Commons CC-BY license (http://creativecommons.org/licenses/by/4.0/). I understand that any comments which I do not wish to be included in my named report can be included as confidential comments to the editors, which will not be published.

I agree to the open peer review policy of the journal

To further support our reviewers, we have joined with Publons, where you can gain additional credit to further highlight your hard work (see: https://publons.com/journal/530/gigascience). On publication of this paper, your review will be automatically added to Publons, you can then choose whether or not to claim your Publons credit. I understand this statement.

Yes
